# Measuring and manipulating localized translation of *erm-1* in the *C. elegans* embryo

**DOI:** 10.1242/dev.204435

**Published:** 2025-05-19

**Authors:** Elise van der Salm, Esther Koelewijn, Mette Schroeder, Erica van der Maas, Olga Jarosińska, Max Eeken, Suzan Ruijtenberg

**Affiliations:** Division of Developmental Biology, Institute of Biodynamics and Biocomplexity, Department of Biology, Faculty of Science, Utrecht University, Padualaan 8, 3584 CH Utrecht, The Netherlands

**Keywords:** Translation, Single-molecule imaging, *C. elegans*, ERM-1

## Abstract

Translation of mRNAs into proteins is key in decoding the information stored in the genome. Localized translation ensures that proteins are expressed where needed, which is important for cell-specific protein expression, the establishment of cellular protein gradients and the creation of protein hotspots. Although localized translation is believed to be important for cell fate determination and organismal development, our understanding of localized translation in the context of living animals is limited, as few methods exist that allow direct visualization and measurement of translation. We adapted the SunTag-based single-molecule translation imaging system for use in *Caenorhabditis elegans*, and show the dynamics and importance of localized *erm-1* translation during development. We found *erm-1* translation to be enriched at the plasma membrane, overlapping with the localization and function of the encoded membrane-cytoskeleton linker ERM-1. Re-localizing *erm-1* translation to nuclear pores disrupts the function of ERM-1 protein, particularly its role in linking the actin cytoskeleton to the membrane, leading to defects in intestinal lumen formation. Our work demonstrates the power of translation imaging and highlights the importance of localized translation in *C. elegans* development.

## Teaser

**Summary:** SunTag-based single-molecule translation imaging in *C. elegans* enables measurement and manipulation of localized *erm-1* translation dynamics, revealing its importance for development.

## INTRODUCTION

Expressing the correct complement of proteins in the right cells at the correct time is crucial for the development of multicellular organisms. Even small changes in expression of a single protein can lead to malfunctioning tissues and disease (Buszczak et al., 2014; Fero et al., 1998; Overton et al., 2014). A key step in achieving accurate protein levels is translation regulation, where the genetic information stored in mRNAs is decoded into functional proteins. Regulation of translation allows temporal and spatially controlled gene expression regulation after transcripts are produced. This is particularly important during developmental transitions and cellular differentiation, when rapid changes in the proteome are required (Sonneveld et al., 2020). As such, several developmental pathways have been identified that control ‘where’, ‘when’ and ‘how efficiently’ mRNAs are translated into proteins.

Localized translation, defined as the synthesis of proteins in specific cells or at specific subcellular regions, is one way to ensure that proteins are expressed exactly where they are needed (Das et al., 2021). It contributes to the establishment of gradients across tissues, the generation of cell type-specific protein expression profiles, and the differentiation of protein composition among different cellular compartments. Localized translation can either be regulated at a global level, influencing all mRNAs within the cell, or at an mRNA-specific level. At the global level, localizing crucial components of the translation machinery can enhance translation efficiency at specific places in the cell. For example, ribosome recruitment to the apical side of intestinal cells in mice results in apically enhanced translation efficiencies upon re-feeding, thereby boosting nutrient absorption (Silva et al., 2022).

mRNA-specific localized translation can be achieved by inhibiting or promoting translation of specific mRNA molecules at particular sites. For example, in *Drosophila melanogaster*, *caudal* mRNA is translationally active only at the posterior side of the embryo due to the asymmetric distribution of the translational repressor Bicoid (Niessing et al., 2002). Similarly, in the early *Caenorhabditis elegans* embryo, *glp-1* mRNAs are present throughout the embryo, but are only translated in the anterior precursor cells. (Evans et al., 1994; Farley and Ryder, 2012; Ogura et al., 2003). Localized translation has also been extensively observed in neurons, where mRNAs needed for the establishment and maintenance of synaptic connections are specifically translated in distal dendrites and axons (Holt et al., 2019; Dalla Costa et al., 2021). Moreover, *actin* is most noticeably translated at the leading edge of migrating fibroblasts (Huttelmaier et al., 2005), and multiple centrosome-associated proteins are found to be translated at the centrosomes (Sepulveda et al., 2018; Safieddine et al., 2021). As such, localized translation corresponds to protein levels and localization, and prevents the accumulation of proteins in undesired cellular places. In addition, localized translation in the right micro-environment has been suggested to be important for the maturation of newly synthesized proteins, facilitating post-translational modifications and the fast assembly of protein complexes (Das et al., 2021).

Localized translation is often intertwined with mRNA localization: if mRNAs are localized to specific sites in the cell, it is highly likely that translation will follow that pattern. However, translation can also drive mRNA localization. Here, the localization signal is provided by the nascent (poly)peptide, rather than the mRNA sequence (Das et al., 2021). This is best described for secreted proteins, where a signal peptide ensures translation at the endoplasmic reticulum (Reid and Nicchitta, 2015). Recently, many more mRNAs have been found to localize during translation via nascent domains that bind cellular structures, guiding mRNA-ribosome complexes to specific locations in the cell (Chouaib et al., 2020; Parker et al., 2020; Winkenbach et al., 2022; Safieddine et al., 2021; Tocchini et al., 2021).

Despite the widespread occurrence of localized translation across organisms, the underlying mechanisms and its importance for animal development remain largely unexplored. For example, it is unclear whether mRNA hotspots result from random diffusion or directed movement, what the time interval is between initiation of translation and localization, and whether mRNA-ribosome complexes are ‘trapped’ or dynamically move between their hotspots and the cytosol. Understanding the importance of localized translation is complicated by the fact that translation-dependent mRNA localization is often guided by a localization signal within the protein, making it difficult to disentangle from protein function. Finally, there is little understanding of when and where proteins are synthesized during development, as there are currently few assays that visualize translation dynamics in living organisms.

Here, we have developed and applied SunTag-based live imaging of translation of single mRNAs in the nematode *C. elegans*, and examined the dynamics and importance of localized translation. We specifically focused on ERM-1, which is the sole ortholog of the human Ezrin/Radixin/Moesin (ERM) family, and plays a key role in organizing the apical cell cortex and lumen formation in tubular epithelia (Gobel et al., 2004; Van Furden et al., 2004). ERM proteins link phospholipids and proteins embedded in the plasma membrane to the underlying actin cytoskeleton. Loss of *erm-1* results in developmental defects and lethality, underscoring its crucial role in animal development (Gobel et al., 2004; Van Furden et al., 2004; Ramalho et al., 2020). Recent research has demonstrated that *erm-1* mRNA co-localizes with ERM-1 protein at the plasma membrane (Parker et al., 2020; Winkenbach et al., 2022; Li et al., 2021). This co-localization depends on translation and the presence of nascent polypeptides, as inhibiting translation or mutation of the membrane-binding domain of ERM-1 diminishes *erm-1* mRNA localization (Winkenbach et al., 2022), suggesting localized translation at the membrane and translation-dependent mRNA localization of *erm-1* (Winkenbach et al., 2022). Using SunTag-based live imaging, we confirm that erm*-1* translation predominantly occurs at the membrane. Localization of *erm-1* translation is highly dynamic and heterogeneous: some translated mRNAs stay in proximity of the membrane for minutes, others lose the interaction and move back into the cytoplasm, even in the presence of translation. This highlights the importance of live tracking this dynamic process and excludes a simple model where every translated mRNA is trapped at the plasma membrane. To assess the functional importance of localized translation, we re-directed *erm-1* mRNA translation to the nuclear envelope using the PP7/PCP tethering system. This mis-localized translation disrupted the apical enrichment of ERM-1, increased its dynamics at the membrane, and interfered with its ability to link the actin cytoskeleton to the apical membrane in intestinal cells, leading to developmental defects in intestinal lumen formation. Together, our data suggest that localized translation of *erm-1* plays a role in its processing, its modification or its ability to form complexes, and that membrane-localized translation contributes to its function in intestinal development. These findings underscore the potential of the SunTag system for investigating translation dynamics in animal development, and emphasize the importance of localized translation and translation-dependent mRNA localization for protein function and development.

## RESULTS

### Visualizing translation of single mRNAs in *C. elegans* embryos and larvae

To visualize the translation of single mRNAs during development, we adapted the microscopy-based SunTag system for use in *C. elegans*. The SunTag live-cell imaging system combines two components that together allow the microscopic detection of nascent polypeptides. The first component is a SunTag peptide array (generally 24xGCN4 peptides) that is incorporated at the 5′-end of a gene of interest. This is combined with co-expression of the anti-GCN4 single-chain variable antibody fragment (scFv) fused to eGFP (scFv::GFP; SunTag antibody). When the mRNA is translated, the GCN4 peptide array emerges from the ribosome first and is rapidly recognized by the cytoplasmic scFv::GFP antibodies. As multiple ribosomes simultaneously translate a single mRNA and each ribosome produces a nascent peptide chain that can be bound by multiple antibodies – up to 24 per nascent peptide chain – this results in the recruitment of numerous antibodies per translating mRNA, which appears as a bright fluorescent spot under the microscope. Although mature proteins are also labeled by the SunTag system, their fluorescence is much dimmer, as each protein can bind no more than 24 antibodies (Ruijtenberg et al., 2020; Tanenbaum et al., 2014; Yan et al., 2016; Wang et al., 2016; Morisaki et al., 2016; Wu et al., 2016; Pichon et al., 2016).

**Fig. 1.**
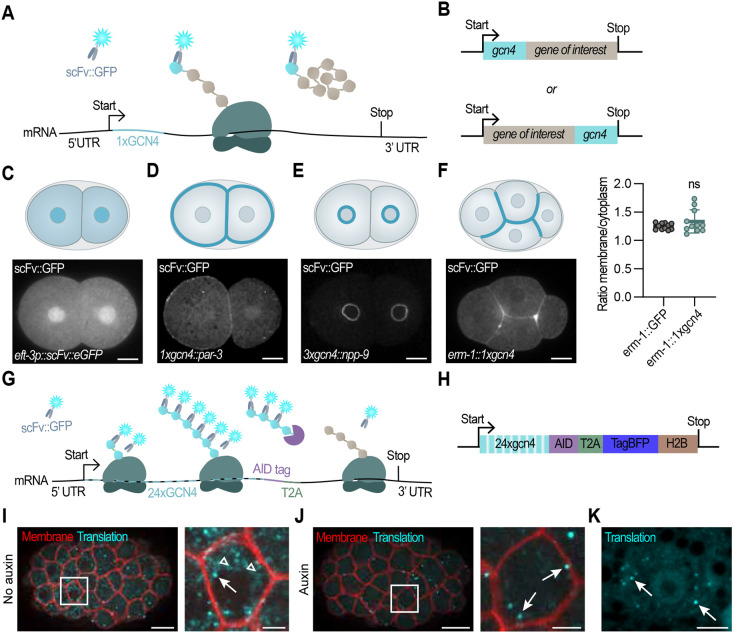
**Visualizing proteins and translation of single mRNAs in *C. elegans* embryos and larvae using SunTag.** (A,B) Schematic overview of the SunTag-based protein-labeling strategy. (C) Schematic overview and representative image of a two-cell stage *C. elegans* embryo expressing the SunTag antibody (scFv::GFP). (D,E) Schematic and representative image of PAR-3 (D) and NPP-9 (E) expression using the SunTag system. (F) Schematic and representative image of ERM-1 expression using the SunTag system (left) and quantification of membrane to cytoplasmic ratio of ERM-1 levels using either GFP or the SunTag system (right). Each dot represents a single embryo (*n*=11). Test of significance: two-tailed unpaired Student's *t*-test with Welch's correction, with significance set at *P*≤0.05. ns, not significant. Scale bars (C-F): 10 µm. (G) Schematic of the translation imaging strategy using the SunTag system. (H) Schematic overview of our translation imaging reporter. (I,J) Representative embryos expressing the SunTag antibody (*scFv::GFP*; depicted in cyan), the translation imaging reporter (*eft-3p::24xGCN4::AID::T2A::BFP::h2b::tbb-2 3′UTR*), the membrane marker (*pie-1p::mCherry::PH*; depicted in red) and TIR protein (*eft-3p::TIR1::F2A::mTagBFP2::AID::NLS*; not shown) without auxin treatment (I) or with auxin treatment (J). Areas outlined in the left images are shown on the right. Arrows indicate translation spots. Arrowheads indicate mature GCN4 proteins. Scale bars: 10 µm (left panel); 2.5 µm (right panel). (K) A representative image of the seam cell plane (skin cells) of an L2 larvae is shown, expressing *scFv::GFP* and the translation imaging reporter *eft-3p::24xGCN4::AID::T2A::BFP::h2b::tbb-2 3′UTR.* Translation spots are indicated by arrows. Scale bar: 5 µm.

To develop the SunTag system for *C. elegans*, we first tested whether the SunTag antibody efficiently binds a single or a few GCN4 SunTag epitopes fused to an endogenous protein (Fig. 1A,B). We expressed a *C. elegans* optimized version of the scFv::GFP SunTag antibody under the control of the ubiquitous *eft-3* promoter (Aljohani et al., 2020; Tanenbaum et al., 2014), resulting in uniform cytoplasmic scFv::GFP expression with slightly higher fluorescence in nuclei of somatic tissues and the germline (Fig. 1C). We then fused the SunTag epitope(s) (19 amino acids and a linker) to the polarity protein PAR-3, the nuclear pore protein NPP-9 and the membrane-cytoskeleton linker protein ERM-1 to assay antibody recruitment. As expected, we observed clear clustering of GFP to the nuclear pores (NPP-9), to the anterior domain of embryonic blastomeres (PAR-3) and to the plasma membrane (ERM-1) (Fig. 1D-F). Membrane enrichment of ERM-1 was comparable between the ERM-1::SunTag and an ERM-1::GFP strain (Fig. 1F, right panel), further confirming that the scFv::GFP antibody can efficiently label GCN4 epitope-tagged proteins in *C.* elegans.

Next, we used the SunTag system to visualize translation. We designed a reporter encoding 24xGCN4 SunTag peptides (∼1.8 kb), followed by BFP::H2B under control of the *eft-3* promoter. This allowed simultaneous visualization of translation (based on the SunTag peptides) and final protein levels (based on BFP::H2B) (Fig. 1G,H). To ensure that the SunTag does not interfere with BFP::H2B expression, we separated the 24xGCN4 SunTag peptides from the BFP::H2B using a T2A ribosome skipping site, resulting in two distinct proteins from the same mRNA in approximately a 1:1 ratio (Fig. 1G,H) (Ahier and Jarriault, 2014). Combined expression of the reporter and the SunTag antibody resulted in the appearance of bright-green fluorescent translation spots in both embryos and larvae, across different tissues (Fig. 1I, arrows). Control embryos solely expressing the SunTag antibody without the SunTag peptides did not exhibit any translation spots (Fig. 1A). Of note, the SunTag reporter is silenced in early embryos and the first translation spots appear around the 16-cell stage. This silencing is caused by the repetitive nature of the 24xSunTag peptide array, as proteins tagged with only one or two SunTag peptides (Fig. 1D-F) are expressed in the early embryo (see also Discussion). In addition to the bright translation spots, we observed numerous dim GFP-positive spots (Fig. 1I, open arrowheads). We hypothesized that these dimmer spots are mature proteins consisting of only one SunTag peptide array. Therefore, we combined the SunTag system with the Auxin Inducible Degron (AID) system (Nishimura et al., 2009; Zhang et al., 2015; Sepers et al., 2022b). The AID system enables targeted degradation of AID-tagged proteins upon addition of auxin and expression of TIR-1, a plant factor that, in complex with auxin, mediates AID recognition by SCF-like E3 ligases. By introducing the AID sequence C-terminally to the SunTag peptides and upstream of the T2A sequence, we specifically degrade the mature SunTag polypeptides without affecting the SunTag signal at the translation sites (Fig. 1G,H) (Wu et al., 2016). Indeed, the dimmer spots largely disappeared upon addition of auxin, indicating that they represent mature proteins rather than translation sites [compare Fig. 1I with Fig. 1J (embryo); Fig. 1K (seam cells)]. Thus, by combining the AID system and the SunTag system we can specifically visualize translation with a high sensitivity.

### Validation of the SunTag system

**Fig. 2.**
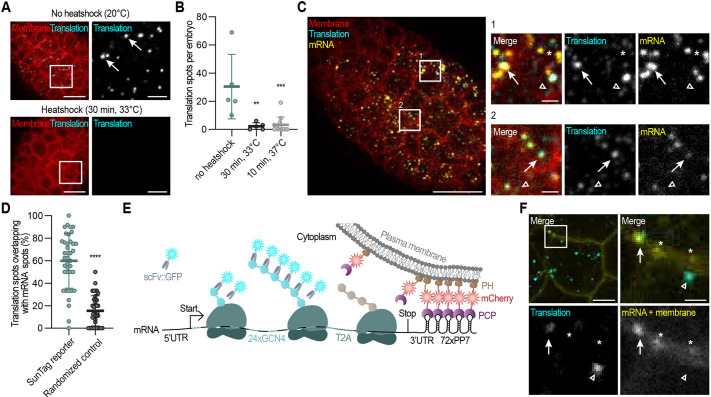
**Validations of the SunTag translation imaging system in *C. elegans.*** (A) Representative embryos expressing the SunTag antibody (*scFv::GFP*), the translation imaging reporter (*eft-3p::24xGCN4::AID::T2A::BFP::h2b::tbb-2 3′UTR*) and the membrane marker (*pie-1::mCherry::PH*), grown at 20°C (top) or subjected to heat-shock (bottom). Areas outlined in the left images are shown on the right. Arrows indicate active translation sites. Scale bars: 10 µm (left); 2.5 µm (right). (B) Quantification of the number of translation spots in 30- to 60-cell embryos in non-treated and heat-shocked animals. Each dot represents a result from one embryo (*n*=5 for no heat shock and 30 min heat shock, *n*=12 for 10 min heat shock). Data are mean±s.d. **P≤0.01, ***P≤0.001 (Kruskal–Wallis test followed by a Dunn's multiple comparisons test versus the ‘no heat shock’ condition). (C) Representative fixed embryo expressing the SunTag antibody (*scFv::GFP*), a translation reporter (*eft-3p::24xGCN4::AID::T2A::BFP::H2B:20xPP7:tbb-2 3′UTR*) and membrane marker (*pie-1p::PCP::mCherry::PH*)*.* Reporter mRNAs (yellow), membranes (red) and translation spots (cyan) were detected using a combination of smiFISH and immunofluorescence. Areas outlined in the left image are shown on the right. Arrows indicate translation spots overlapping mRNA. Arrowheads indicate mature GCN4 proteins (weak cyan signal) that do not overlap mRNAs. Asterisks indicate non-translated mRNA. Scale bars: 10 µm (large panel); 1 µm (small panels). (D) Quantification of the number of bright cyan spots (presumable translation spots) that overlap mRNA spots. Each spot represents one embryo (*n*=39 for both treatments). Data are mean±s.d. ****P≤0.0001 (two-tailed unpaired Student's *t*-test with Welch's correction). (E) Schematic overview of the SunTag translation imaging system in combination with mRNA labeling and membrane tethering using the PP7 system. (F) Representative image of part of a live embryo, extra-chromosomally expressing the reporter, as shown in C, indicating overlap between translation spots (cyan) and mRNAs (yellow). The area outlined in the top-left image is shown in the three other images. Arrows indicate translation spots overlapping mRNA. Open arrowheads indicate translation spots not overlapping mRNA. Asterisks indicate non-translated mRNAs. Scale bars: 2.5 µm (top left); 0.75 µm (top right and bottom row). See also Fig. S1.

To validate whether the bright GFP spots are active sites of translation, we made two assumptions: (1) the spots should disappear upon inhibition of translation; and (2) they should co-localize with mRNAs. When we inhibited translation, by subjecting the animals to a short heat shock, we observed a rapid and significant reduction in the number of translation spots, in support of our first assumption (Fig. 2A,B). Next, we assessed co-localization between individual mRNAs and presumed translation spots, in both fixed and live embryos. In fixed samples, we combined single-molecule inexpensive fluorescence *in situ* hybridization (smiFISH) to visualize reporter mRNA with immunofluorescence-based detection of the scFv::GFP to visualize translation (Fig. 2C,D) (Tocchini et al., 2021). We observed that ∼60% of the bright GFP clusters coincide with an mRNA spot, supporting our second assumption (Fig. 2D). While most translation spots overlapped with mRNA, a substantial fraction did not. This could have two possible explanations. First, some identified bright GFP spots may not be active translation sites, but mature proteins or protein aggregates that met our analysis threshold. Second, this may reflect a low labeling efficiency of our mRNA probes (only ∼62%; see Materials and Methods, Fig. S1A) and consequent failure to detect a fraction of reporter mRNA molecules. Together, this indicates that most or all GFP spots indeed represent active translation. Interestingly, in line with recent literature, we also observed that many mRNAs are not translated (Sonneveld et al., 2020), highlighting the need for single-molecule measurements to explore translation heterogeneity.

Next, we set out to visualize mRNA and translation simultaneously in live animals. To achieve this, we combined the SunTag system with the PP7/PCP system for mRNA detection (Chao et al., 2008; Li et al., 2021). This involved introducing 72 copies of a PP7 hairpin sequence into the 3′UTR of our reporter gene and co-expressing the PP7 bacteriophage coat protein (PCP) fused to mCherry and a PH domain (PCP::mCherry::PH). The PCP::mCherry::PH proteins localize to the membrane and bind with high affinity to the PP7 hairpin sequences, thereby tethering the reporter mRNAs to the plasma membrane, reducing their mobility and facilitating long-term tracking of individual mRNA molecules. The 72xPP7 array in the mRNA clusters the PCP::mCherry::PH signal, which allows us to observe mRNAs as individual mCherry-fluorescence spots (Fig. 2E). Using this approach, we often observed the overlap between mRNA and translation spots (Fig. 2F). However, we also observed translation spots that did not overlap with mRNA, which may be due to a low mRNA detection by the PP7/PCP system in *C. elegans* or to low PCP::mCherry::PH levels. Interestingly, tracking of membrane-tethered SunTag mRNAs over time revealed the separation of a bright GFP spot that stayed at the membrane and a dim GFP spot that diffused away. This dim spot is most likely an individual, fully synthesized SunTag protein, released from the translation site (bright spot) (Fig. S1B). Together, these results show that the bright GFP SunTag spots are sites of active translation, and highlight its powerful potential to study translation dynamics of single mRNAs in real-time in live *C. elegans* embryos and larvae.

### Localized translation of endogenous *erm-1* mRNA

**Fig. 3.**
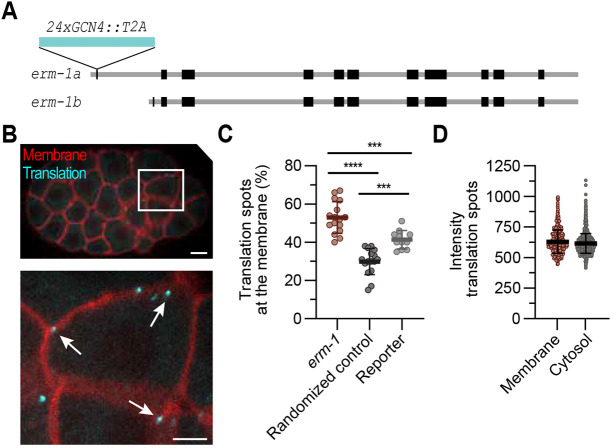
**Translation of *erm-1* is enriched at the plasma membrane.** (A) Schematic of the *erm-1* locus with *erm-1a* and *erm-1b* isoforms, and insertion of 24xGCN4 peptides. (B) Representative embryo expressing *24xGCN4::T2A::erm-1*, the SunTag antibody (scFv::GFP) and the membrane marker (mCherry::PH). Membranes are in red; translation sites are in cyan. The area outlined in the top image is shown in the image below. Arrows indicate translation sites in close proximity to the membrane. Scale bars: 10 µm (top); 5 µm (bottom). (C) Quantification of the number of translation spots within 0.35 µm of the membrane in the *24xGCN4::T2A::erm-1* strain, in a randomized computed control (see Materials and Methods section ‘Quantification of localized translation’) and in the *eft-3p::24xGCN4::AID::T2A::BFP::h2b::tbb-2 3′UTR* strain. Each dot represents the percentage of spots in the proximity of the membrane in a single embryo (*n*=16 for *erm-1* and randomized control, *n*=11 for reporter). ***P≤0.001 and ****P≤0.0001 (one-way ANOVA with Bonferroni's post-hoc test). (D) Quantification of the intensity of translation spots at the membrane and in the cytosol. Each dot represents a single translation spot (*n*=480 for membrane, *n*=1744 for cytosol). See also Fig. S2.

In the *C. elegans* embryo, localized translation has been suggested for several polarized proteins, including DLG-1, AJM-1 and ERM-1 (Parker et al., 2020; Winkenbach et al., 2022; Tocchini et al., 2021; Tocchini and Mango, 2024). We applied the SunTag system to study the dynamics of localized *erm-1* translation in developing embryos. Similar to our reporter, we inserted 24 SunTag peptides followed by a T2A sequence between the start codon and coding sequence of endogenous *erm-1a* (Fig. 3A), to allow visualization of translation directly from the start of the process. We combined this with expression of the scFv::GFP SunTag antibody and a membrane marker (*pie-1p::mCherry::PH*) (*erm-1^24xSunTag^* strain). The T2A sequence is important because N-terminal protein fusion of ERM-1 can interfere with its function (Roy et al., 1997; Henry et al., 1995; Amieva et al., 1999). We observed a slightly lower growth rate and brood size (Fig. S2A,B), but no developmental or morphological defects related to loss of *erm-1* in our *erm-1^24xSunTag^* strain. We first examined *erm-1^24xSunTag^* mRNA levels using smiFISH. In contrast to our *erm-1::GFP* control, *erm-1^24xSunTag^* transcripts could not be observed in the early embryos, which is probably due to germline silencing. However, multiple *erm-1^24xSunTag^* transcripts appeared between the 16- and 32-cell stage, and expression was abundant during later stages. From the 40-cell stage, there was no difference in either the number or location of *erm-1* transcripts between the *erm-1::GFP* control and *erm-1^24xSunTag^* animals (Fig. S2C). Next, we examined translation of *erm-1^24xSunTag^* using live imaging. The first translation spots became visible from the 32-cell stage onward, consistent with the observed mRNA expression patterns. Around the 64-cell stage, multiple translation spots emerged at the plasma membrane, across the entire embryo (Fig. 3B). By the bean stage, translation spots were visible in different cell types, including the intestinal progenitor cells (Fig. S2D), one of the tissues where ERM-1 is highly expressed and where its function is crucial (Ramalho et al., 2020). Taken together, we created an endogenously SunTag-tagged *erm-1* strain, which allows us to study translation localization of *erm-1a* over the course of development, except for the earliest stages of embryonic development.

To determine whether translation is indeed enriched at the membrane, we quantified the percentage of spots that resided within a 0-0.35 μm distance from the membrane. We found an average of 53% (ranging from 40% to 67% in individual embryos) of translation spots localized at the membrane, which is significantly more than either our SunTag BFP::H2B reporter (41% resided within the 0-0.35 μm range) or a computed Z-flipped control (30% of the spots resided within 0-0.35 μm range) (Fig. 3C). No clear difference in spot intensity was observed between *erm-1* translation at the membrane or in the cytoplasm (Fig. 3D). Noteworthy, the 53% enrichment in *erm-1* translation at the membrane is higher than the 40% membrane enrichment of *erm-1* mRNA observed by the Nishimura lab, which fits the model that translated mRNAs have an increased chance of being located close to the membrane (Winkenbach et al., 2022).

In *C. elegans* embryonic blastomeres, ERM-1 protein localizes to the entire plasma membrane. However, in mid-embryonic stages, when intestinal cells undergo polarization and the apical domain is formed, ERM-1 protein becomes almost exclusively localized to the apical membrane (Van Furden et al., 2004; Gobel et al., 2004). This apical localization of ERM-1 is hypothesized to occur in a two-step process. First, ERM-1 proteins localize to membranes in general (because of FERM domain interactions with PIP_2_) and, second, phosphorylation of ERM-1 stimulates the specific apical localization (Yonemura et al., 2002; Fievet et al., 2004; Ramalho et al., 2020). Based on mRNA localization patterns in control and ERM-1 phosphorylation-defective mutants, it has been suggested that the movement of ERM-1 protein from the basolateral to the apical membranes occurs post-translationally (Winkenbach et al., 2022). Indeed, in mid-stage embryos there is an enrichment of translation at both the lateral and apical membrane (Fig. S2D). This demonstrates that *erm-1* is translated near the membrane, but not exclusively at the apical site, where ERM-1 function is required for apical membrane morphogenesis and tube formation.

### Dynamics of localized translation

**Fig. 4.**
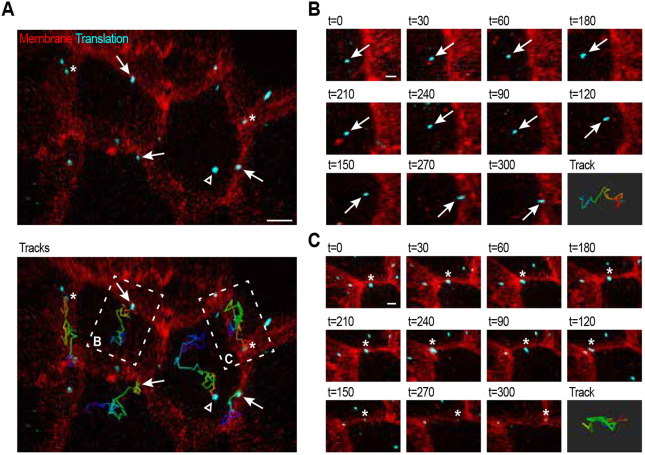
***erm-1* translation spots show different dynamic behaviors.** (A) Live tracking of individual translation sites in animals expressing *24xGCN4::T2A::erm-1*, the SunTag antibody (scFv::GFP) and the membrane marker (mCherry::PH). Translation spots are in cyan, membranes are in red. Bottom panel shows the movement tracks of individual spots over time. Dark blue is the start, red the end of the track. Arrows indicate translation spots starting in the cytoplasm and localizing to the membrane. Arrowheads indicate translation spots starting and staying in the cytoplasm. Asterisks indicate translation spots located in the vicinity of the membrane from start to finish. (B,C) Time series of individual translation spots in A. Displayed track is based on all images (every 5 s) from the recording. Arrows and asterisks indicate the translation spots. Scale bars: 2.5 µm in A; 1 µm in B and C. See also Movie 1.

The current model of localized *erm-1* translation (Parker et al., 2020; Winkenbach et al., 2022; Li et al., 2021) proposes that *erm-1* mRNAs rapidly locate to the membrane upon translation initiation. However, this is a hypothesis, as visualization of *erm-1* translation was not feasible before, and the dynamics and heterogeneity of translated mRNAs are unknown. Using timelapse imaging and single-molecule tracking of *erm-1* translation spots, we identified a variety of different mRNA translation behaviors (Fig. 4A and Movie 1). First, we observed *erm-1* translation events that started in the cytoplasm, and over time localized to the membrane, where they then remained for the duration of the recording (5 min) (Fig. 4A, arrows; Fig. 4B), in line with the proposed model. Second, we found translated mRNAs that moved throughout the cytoplasm without interacting or staying close to the membrane, indicating that localization can take longer than 5 min or that translation does not always lead to localization to the membrane (Fig. 4A, open arrowhead). Given that 5 min should be sufficient for ribosomes to have synthesized the PIP_2_-binding domain from *erm-1^24xSunTag^* mRNAs, this suggest that not every nascent peptide will interact with the membrane, preventing mRNA localization to the membrane (see Discussion). Third, we observed mRNAs that were already near the membrane at the start of the recording and stayed there for the entire 5 min duration (Fig. 4A, asterisks; Fig. 4C). Despite their localization near the membrane, these mRNAs were highly mobile, moving laterally across the membrane. This lateral movement may be due to membrane fluidity or to constant interaction and dissociation between PIP_2_ and ERM-1. Furthermore, this could indicate multiple rounds of translation where one nascent protein is released before the next nascent FERM domain binds PIP_2_.

We observed both non-directed movements (e.g. blue part, translation track, Fig. 4B), as well as rapid directional movements of *erm-1* translation (e.g. green part, translation track, Fig. 4B). These switches between movement categories have been reported for *erm-1* mRNA before, based on live imaging of *erm-1* reporter mRNAs, and were attributed to transitions between a ribosome-free and a ribosome-bound state (Li et al., 2021). However, our data indicate that, even in the presence of translating ribosomes, the speed and directionality of mRNA movement changes over time.

### Localized translation of *erm-1* is important for ERM-1 function

**Fig. 5.**
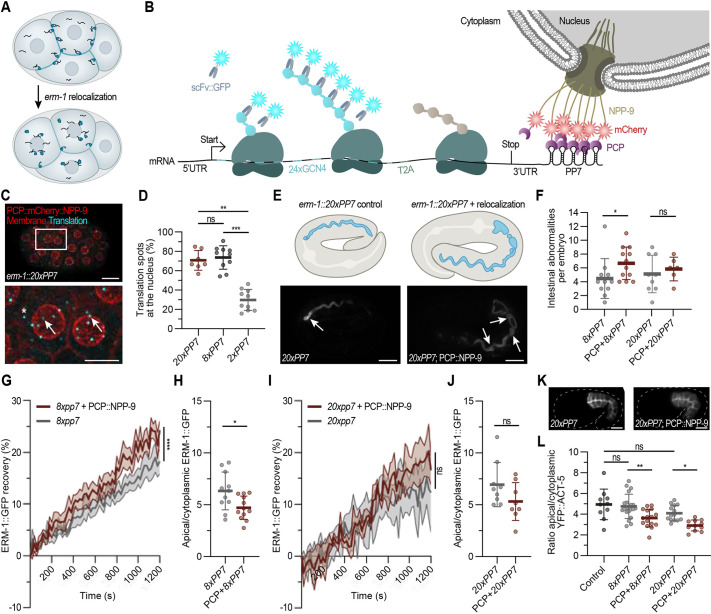
**Disruption of localized *erm-1* translation affects ERM-1 protein function.** (A) Schematic overview of re-localizing *erm-1* mRNAs from the membrane to the nuclear pore. (B) Schematic overview of SunTag-based translation imaging of *erm-1* in combination with the PP7 system. (C) A representative embryo expressing *24xGCN4::T2A::erm-1::20xPP7*, PCP::mCherry::NPP-9 (red) and mCherry::PH (red). The area outlined in the top image is shown in the image below. Arrows indicate spots at the nucleus. Asterisk indicates a cytoplasmic spot. Scale bars: 10 µm (top); 5 µm (bottom). (D) Quantification of re-localization efficiency using 20xPP7 (*n*=7), 8xPP7 (*n*=10) or 2xPP7 (*n*=10). Data are mean±s.d. **P≤0.01, ***P≤0.001 (Kruskal–Wallis test with Dunn's multiple comparisons test). ns=not significant. (E) Representative images of embryos expressing YFP::ACT-5. Arrows indicate intestinal defects in embryos with (right) or without (left) *erm-1* re-localization. Scale bars: 10 µm. (F) Quantification of the number of intestinal abnormalities (twists, constrictions and cysts) in embryos with or without *erm-1* re-localization. Each dot represents one embryo (*n*=11, 12, 9, 6). Data are mean±s.d. *P≤0.05 (Kruskal–Wallis test with Dunn's multiple comparisons test). ns=not significant. (G-J) Quantification of ERM-1 dynamics at the membrane by FRAP in L1 larvae with or without *erm-1* re-localization (*n*=7 for 8xPP7, *n*=5 for 8xPP7+PCP, *n*=3 for 20xPP7, *n*=3 for 20xPP7+PCP) (G,I), and ERM-1 enrichment at the membrane in embryos with or without *erm-1* re-localization (*n*=11 for 8xPP7, *n*=12 for 8xPP7+PCP, *n*=9 for 20xPP7, *n*=8 for 20xPP7+PCP) (H,J). Data are mean±s.d. *P≤0.05, ****P≤0.0001 (FRAP data: RM two-way ANOVA with Geisser-Greenhouse correction; apical ERM-1 enrichment: two-tailed unpaired Student's *t*-test). ns=not significant. (K) Intestinal distribution of YFP::ACT-5 in embryos with (right) or without (left) *erm-1* re-localization. Scale bars: 10 µm. (L) Quantification of the apical to cytoplasmic ratio of YFP::ACT-5 levels. Each dot represents one embryo (*n*=10 for control, *n*=20 for 8xPP7, *n*=16 for 8xPP7+PCP, *n*=13 for 20xPP7, *n*=11 for 20xPP7+PCP)*.* Data are mean±s.d. *P≤0.05, **P≤0.01 (Kruskal–Wallis test with Dunn's multiple comparisons test). ns=not significant. See also Fig. S3.

Localized translation of *erm-1* could be functionally important as it may regulate ERM-1 expression, localization or post-translational modifications. Alternatively, it could be a by-product of co-translational binding of nascent ERM-1 proteins to the plasma membrane via the PIP_2_-binding domain, dragging the mRNA-ribosome complex along. It has been demonstrated that altering ERM-1 protein localization or function affects *erm-1* mRNA localization at the membrane (Winkenbach et al., 2022). Here, we ask the reverse: if we alter the cellular site where ERM-1 is synthesized, does this impact ERM-1 protein localization and/or function? For this purpose, we used the PP7/PCP mRNA tethering system to re-localize *erm-1* mRNAs to nuclear pores, relatively distant from the plasma membrane (Fig. 5A,B).

We first tested whether the PP7/PCP system can efficiently direct mRNA localization to either the membrane or nuclear pores. We incorporated 20xPP7 hairpins into the 3′ UTR of our SunTag::BFP::H2B reporter and co-expressed PCP fused to the membrane (PCP::mCherry::PH) or nuclear pore protein 9 (NPP-9) (PCP::mCherry::NPP-9). Translation spots efficiently re-localized to both the membrane (89%) and nuclear pores (80%), respectively (Fig. S3A,B). The total number of translation spots decreased upon inserting PP7 sites, consistent with the idea that inserting PP7 hairpins triggers mRNA decay (Fig. S3C) (Tocchini and Mango, 2024), but were similar across PP7-sites containing strains. Notably, the intensity of translation spots was higher at the nuclear pore compared to either cytoplasmic translation (PP7 alone) or when tethered to the membrane (PP7+PCP::mCherry::PH) (Fig. S3D). Consistent with this, BFP expression levels were elevated when mRNA was tethered the nuclear pore, suggesting that the environment around the nuclear pores is favorable for active translation (Fig. S3E,F). Importantly, tethering mRNAs did not cause developmental defects, indicating that our system efficiently re-localizes mRNAs without affecting overall animal development.

Next, we applied our tethering technique to *erm-1.* Since large PP7 insertions can trigger nonsense-mediated decay (Tocchini and Mango, 2024), we analyzed strains with varying PP7 site numbers. We inserted 20xPP7, 8xPP7 or 2xPP7 arrays into the 3′ UTR of *erm-1^24xSunTag^*, creating *erm-1^24xSunTag;PP7^* strains in which all *erm-1* mRNAs contain PP7 sites. *erm-1* mRNAs with 20x or 8xPP7 were efficiently re-localized to the nuclear pore when combined with PCP::mCherry::NPP-9 expression (71% and 74%, respectively) (Fig. 5C,D). However, 2xPP7 was insufficient to re-localize *erm-1* and was thus excluded from further analysis (Fig. 5D). Nuclear pore-localized *erm-1* mRNAs were efficiently translated, as re-localized *erm-1^24xSunTag;PP7^* strains showed slightly higher number and intensity of translation spots compared to non-localized *erm-1^24xSunTag;PP7^* control strains (Fig. 3G,H). To assess the impact of PP7 insertions on ERM-1 expression, we introduced PP7 hairpins into an *erm-1::GFP* strain. Both 20xPP7 and 8xPP7 strains reduced ERM-1::GFP expression, probably due to the elongated 3′UTR that triggers mRNA decay (Fig. S3I). In line with this, ERM-1 levels were more affected in the 20xPP7 strain than in the 8xPP7 strain. Importantly, in 8xPP7 strains, ERM-1 levels remained comparable to heterozygous *erm-1::GFP* animals, which is sufficient for normal development. Interestingly, in line with increased translation efficiency at the nuclear pore (Fig. S3G,H) and reporter observations (Fig. S3C-F), *erm-1* re-localization restored ERM-1::GFP levels closer to wild type in 8x and 20xPP7 larvae, and 20xPP7 embryos (Fig. S3I,J). However, this effect was absent in 8xPP7 embryos (Fig. S3I). The underlying cause remains unclear but may be related to increased ERM-1 dynamics when synthesized at the nuclear pore (see below). Overall, while ERM-1 expression is affected, it levels appears sufficient for normal development, especially in the 8xPP7 strains, for studying the functional consequences of disturbed localized translation.

To assess the functional consequences of *erm-1* re-localization, we examined developmental defects in re-localizing *erm-1* mutants (Fig. 5F, Fig. S3K,L). Strikingly, embryos in which *erm-1* was translated at the nuclear pore showed several developmental defects, resembling *erm-1* mutants, including cysts and constrictions in the intestinal lumen (Fig. 5F). In contrast, control 8xPP7, non-re-localized strains, exhibited significantly fewer abnormalities (Fig. 5F), indicating that normal, membrane-localized translation of *erm-1* is functionally relevant. As these functional consequences are probably caused at the molecular level, we studied how localized translation affects ERM-1 localization and dynamics, and its ability to link the actin cytoskeleton to the membrane.

Accurate ERM-1 localization at the membrane is necessary for ERM-1 in linking the actin cytoskeleton to the apical membrane of epithelial cells. In *8xPP7 erm-1* re-localized strains, we observed significantly reduced apical enrichment of ERM-1::GFP (Fig. 5H,J). This is not due to lower translation efficiency (Fig. S3G-J). Instead, our findings suggest that *erm-1* translation at the membrane contributes to apical targeting of ERM-1. One possible explanation for the reduced apical enrichment of ERM-1 is increased ERM-1 dynamics at the membrane. (Ramalho et al., 2020). Fluorescence recovery after photobleaching (FRAP) experiments indeed revealed faster recovery speed of ERM-1::GFP in *erm-1* re-localized strains compared to PP7 control strains, indicating increased ERM-1 dynamics when synthesized at the nuclear pore (Fig. 5G,I). Although stronger, a similar trend was found in phosphorylation mutants, where reduced apical enrichment correlates with increased ERM-1 turnover at the membrane (Ramalho et al., 2020). Notably, ERM-1 apical enrichment and dynamics were significantly increased in 8xPP7 strains, while the 20xPP7 showed a similar trend, although without reaching statistical significance, probably due to the overall more severe impact of the 20xPP7 integration or lower sample size.

Finally, we aimed to address the ability of ERM-1 to link the actin cytoskeleton to the apical membrane. The apical actin network in *C. elegans* intestinal cells mainly comprises the specialized actin ACT-5 (MacQueen et al., 2005). The integrated transgene YFP::ACT-5 has been used previously to assess actin distribution in *erm-1* mutant animals. Here, loss of ERM-1 function resulted in the loss of YFP::ACT-5 from the apical membrane, accompanied by elevated levels of cytoplasmic and basolateral actin (Ramalho et al., 2020; Sepers et al., 2022a; Hipfner et al., 2004). Therefore, we analyzed whether ACT-5 distribution is affected by re-localizing *erm-1* to nuclear pores. As expected, we detected ACT-5 enrichment at the apical domain of intestinal cells in both wild-type and PP7 control mid-stage embryos. In contrast, in *erm-1* re-localization strains, ACT-5 was significantly diminished at the apical cortex and elevated in the cytoplasm (Fig. 5K,L). These data suggest that localized translation of ERM-1 is important for the recruitment of actin to the apical membrane.

Taken together, our findings demonstrate that shifting *erm-1* translation from the membrane to the nuclear pore significantly disrupts ERM-1 localization, stability and function. While PP7 site insertion reduced overall ERM-1 levels and causes some general defects (Fig. S3I,M,N), altering the translation site leads to defects in membrane targeting, increased ERM-1 turnover and impaired ACT-5 recruitment. This supports a model in which the cellular site of translation directly contributes to ERM-1 behavior, leading to functional consequences for lumen formation.

## DISCUSSION

Here, we report the development, validation and use of the SunTag translation imaging system in *C. elegans*. This system enables live imaging of single-molecule translation events, providing a framework to understand translation regulation in *C. elegans* development. Using this approach, we followed translation of individual mRNAs live and detected localized translation for the membrane-cytoskeleton linker protein ERM-1. Our findings suggest that membrane-localized *erm-1* translation contributes to the function of ERM-1 in recruiting actin to the apical membrane, which is important for intestinal development and morphology.

### The SunTag system as a general tool for studying translation and visualizing proteins of interest

Using the SunTag system, we obtained unique insights in the translation dynamics of *erm-1*. In addition, the system has broad potential for studying various aspects of *C. elegans* development where mRNA translation localization and efficiency are important, including neuronal development, cell cycle regulation and early embryogenesis (Das et al., 2021). Beyond translation imaging, SunTag enables visualization of proteins of interest. Due to its small size of only 19 amino acids, multiple SunTag peptides can easily be fused to genes of interest, amplifying fluorescent signals. Fluorescence amplification can be of great benefit for single molecule imaging (Hu et al., 2023) or visualization of lowly expressed proteins. Moreover, SunTag can be used to visualize highly dynamic proteins (e.g. transcription factors), which are challenging to observe using conventional fluorophores because of long maturation times (Bothma et al., 2018; Chang and Dickinson, 2022; Tanenbaum et al., 2014). The SunTag system overcomes this issue as the pre-maturated SunTag antibodies immediately visualize proteins that are fused to the SunTag peptides (Dufourt et al., 2021; Vinter et al., 2021).

### Considerations when applying the SunTag system and PP7/PCP system in *C. elegans*

Visualizing mRNAs and their translation provides valuable quantitative information on post-transcriptional gene regulation. However, both the PP7/PCP and SunTag-systems require genetic manipulation, potentially affecting expression of target genes. For example, integrating the SunTag array into the endogenous locus of *erm-1* resulted in germline silencing. Reducing the number of SunTags, removing piRNA sites from the SunTag sequences (Wu et al., 2018; Zhang et al., 2018) or introducing *smu-1* introns (Aljohani et al., 2020) may help overcome germline silencing in the future. Similarly, introducing PP7 hairpins in the 3′UTR of *erm-1* reduced ERM-1 expression (Fig. S3I,J), probably because longer UTRs are more prone to be recognized and degraded by the nonsense-mediated decay machinery (Buhler et al., 2006; Eberle et al., 2008; Hogg and Goff, 2010). Changing the location of hairpins could reduce this effect, as demonstrated previously for *dlg-1* (Tocchini and Mango, 2024). Finally, when using the SunTag system in *C. elegans*, it is important to note that the scFv::GFP SunTag antibody has some affinity to structures close to epithelial apical junctions (Fig. S4A,B) (Hu et al., 2023). Furthermore, the mature GCN4 proteins have affinity for structures near the DNA of mitotic cells (Fig. S4C), but this is prevented by fast degradation of GCN4 proteins using the AID system. In conclusion, the SunTag system is a powerful method for measuring translation dynamics in *C. elegans*, and specific optimization could further enhance its utility in studying translation across all tissues and developmental stages.

### The importance of *erm-1* translation at the membrane for ERM-1 function

In other species, localized translation has been suggested to be important in creating local hotspots of protein concentration or for protein maturation, processing and post-translational modification (Das et al., 2021; Kim et al., 2013). Our data show that translating *erm-1* at the nuclear pore instead of the membrane alters ERM-1 behavior. Specifically, ERM-1 becomes more dynamic at the membrane, less enriched at the apical membrane and less effective in linking the actin cytoskeleton to the membrane. These changes collectively lead to defects in intestinal lumen formation, closely resembling phosphorylation mutants. Phosphorylation of ERM-1 generally occurs after membrane binding and is important for maintaining an open conformation that enables ERM-1 to link the membrane and actin cytoskeleton (Yonemura et al., 2002; Fievet et al., 2004; Ramalho et al., 2020). We speculate that membrane-localized translation facilitates rapid phosphorylation, ensuring that ERM-1 proteins are in the ‘ON’ state, ready to function. This may occur either because ERM-1 remains in an open conformation by immediate membrane binding during translation (Winkenbach et al., 2022) or by co-enrichment of the ERM-1 kinase at the membrane. However, the specific kinase responsible for ERM-1 phosphorylation in *C. elegans* remains unidentified at this stage, leaving the question of which kinase requires *erm-1* to be translated near the membrane for efficient phosphorylation open for investigation. In case *erm-1* is not translated close to the membrane, as in our *erm-1* re-localization strains, the N-terminal FERM domain and C-terminal C-ERMAD domain of ERM-1 probably form an intermolecular interaction, promoting an inhibited state that remains cytoplasmic and unable to function (Gary and Bretscher, 1995; Li et al., 2007; Magendantz et al., 1995; Pearson et al., 2000), potentially lowering ACT-5 levels at the apical membrane.

While most *erm-1* mRNAs are translated at the membrane, a substantial fraction is translated in the cytoplasm, raising the question of why translation at the membrane is not important for these mRNAs. One possibility is that early embryogenesis requires only a modest (∼1.3-fold) enrichment of ERM-1 at the membrane (Fig. 1F), suggesting either an excess of ERM-1 or that phosphorylation is not yet critical – consistent with the lack of defects in *erm-1* phosphorylation mutants at this stage (Ramalho et al., 2020). Localized translation could become essential later, during intestinal differentiation and polarization around the bean stage, when ERM-1 starts to localize primarily apically (Gobel et al., 2004; Van Furden et al., 2004). At this stage, localized translation would ensure fast activation of ERM-1, supporting its specialized role in apical membrane expansion. While some ERM-1 proteins produced in the cytoplasm may eventually be phosphorylated, this process might be too slow to meet the demands of the rapidly developing embryo.

### Heterogeneity in *erm-1* translation

Although all ERM-1 proteins contain a PIP_2_-binding domain, not all translated mRNAs localize to the membrane. One potential explanation for this discrepancy is technical limitations. Our method requires at least two simultaneously translating ribosomes per transcript, since the T2A sequence separates the SunTag peptides encoded at the 5′-end (required for visualization) from the ERM-1 PIP_2_-binding domain (which is required for membrane localization). Therefore, if only downstream ribosomes are present, mRNAs may localize to the membrane, without being detected. Of note, we did not observe disappearance of signal from membrane-localized mRNAs within our 5-min timelapse, indicating that translation was efficient enough to maintain at least two ribosomes on the transcript. Conversely, if ribosomes are present only at the SunTag peptide sequence, translation may be visible without localization. Although some mRNAs might still be in early translation stages, ribosome elongation speeds (ranging from 3-18 codons/s) (Dao Duc and Song, 2018; Ingolia et al., 2011; Yan et al., 2016; Wu et al., 2016; Wang et al., 2016; Pichon et al., 2016; Morisaki et al., 2016) suggest that most ribosomes should reach the PIP_2_ domain (938 codons downstream of the start: 640 codons for the SunTag array and T2A site, 298 codons for *erm-1*) within our 5 min imaging time window. Although we lack real-time elongation measurements, and we cannot rule out the possibility of ribosomes elongating very slowly; slow elongation or pausing seems unlikely due to our optimized codon usage. Taken together, while technical limitations may underestimate the proportion of membrane-localized translation events, this is unlikely the sole explanation for the presence of cytoplasmic translation spots, suggesting biological heterogeneity in mRNA localization.

Translated *erm-1* mRNAs that remain cytoplasmic may either fail to be transported, or do not stabilize at the membrane. However, since most *erm-1* mRNAs stay membrane-bound during translation (Fig. 4A,C), de-stabilization is unlikely to be the main cause. Localization of *erm-1* mRNAs to the membrane is thought to be mediated by dynein in a translation-dependent manner (Li et al., 2021). One possibility is that only the most efficiently translated mRNAs, with many nascent ERM-1 domains, are transported. However, this seems unlikely, as SunTag signal intensity does not differ between cytosolic and membrane-bound translation spots (Fig. 3D). Alternatively, recognition and translocation of the *erm-1* mRNA-ribosome complexes is time-consuming, as timelapse imaging shows that localization to the membrane can take several minutes. The exact mechanisms and required adapters remain unknown, raising key questions for future research: What causes the heterogeneity in membrane localization, and why are some mRNAs recognized and transported while others are not, despite both undergoing active translation.

## MATERIALS AND METHODS

### *C. elegans* strains and culture conditions

All strains were maintained according to standard methods at 15°C or 20°C (Brenner, 1974), unless stated otherwise. Animals were grown on nematode growth medium (NGM) agar plates seeded with OP50 *Escherichia coli* (*E. coli*). A complete list of strains used in this study can be found in Table S1.

### *C. elegans* strain generation

Details on the generation of individual strains, derived from various backgrounds and approaches, are provided in Table S2. For stable single copy integration of the SunTag antibody and reporter at MosSCI loci, a plasmid-based CRISPR/Cas9 method was used (Friedland et al., 2013). Injection mixes contained *eft-3p::Cas9* plasmid (50 ng/μl; Addgene plasmid #46168), single guide RNA (sgRNA) plasmid (50 ng/μl), repair template plasmid (50 ng/μl) and co-injection marker plasmid (2.5 ng/μl) (sgRNAs and repair templates used are listed in Table S2). Offspring expressing the co-injection marker were selected, lysed and genotyped to identify genome-edited worms. For integrations at endogenous loci or SunTag reporter alleles, a Cas9 ribonucleoprotein-based approach was used (Ghanta et al., 2021). Injection mixes contained Cas9 protein (0.25 μg/μl) (IDT), TracRNA (0.1 μg/μl) (IDT), pRF4 [rol-6(su1006)] (40 ng/μl) co-injection marker (Mello et al., 1991), locus-specific crRNA (56 ng/μl) (IDT) and either ssODN (0.11 μg/μl) (IDT) or melted dsDNA (25 ng/μl) repair template (crRNAs and repair templates are listed in Table S2). dsDNA repair templates were amplified by PCR using primers with containing 5′ SP9 modifications (IDT) and purified using the NucleoSpin Gel and PCR Clean-up Kit (Macherey-Nagel, 740609.250) prior to injections. For extrachromosomal array generation, animals were microinjected with λ-DNA (50 ng/μl) (Thermo Fisher), repair template plasmid (20 ng/μl) and co-injection marker (2.5 ng/μl) (used plasmids sequences are listed in Table S2). Progeny expressing the co-injection marker were selected. Correct genome editing or presence of extrachromosomal arrays was confirmed by PCR and Sanger sequencing (Macrogen Europe). For strains generated by crossing, males were obtained by culturing of L4 hermaphrodites at 30°C for 6 h, followed by incubation at 20°C for 3 days. These males were backcrossed with hermaphrodites of the same strain and maintained according to standard procedures, before crossing with other strains.

### Molecular cloning

The scFv::GFP was based on the pHR-scFv-GCN4-sfGFP-GB1-dWPRE plasmid (Addgene plasmid #60907) (Tanenbaum et al., 2014) and germline optimized (Aljohani et al., 2020). To create the repair template plasmids for the SunTag reporter integrations, 24xGCN4 sequences were derived from the plasmid pcDNA4TO-24xGCN4_v4-kif18b-24xPP7 (Addgene plasmid #74928) (Yan et al., 2016). The 24xGCN4 sequence consists of 24 GCN4 peptides (19 amino acids per peptide, amino acid sequence EELLSKNYHLENEVARLKK), which are separated by linker sequences (five amino acids, amino acid sequence GSGSG), with a total length of ∼1.8 kb. PP7 hairpin sequences were a kind gift from the Tanenbaum lab (Hubrecht Institute, Utrecht) and the PCP-coding sequence was a kind gift from the Galli Lab (Hubrecht Institute, Utrecht). Primers and G-blocks used for molecular cloning were ordered from IDT. Vectors and DNA fragments were either digested by restriction enzymes (Thermo Scientific or NEB) and ligated by T4 ligase (NEB M0202L) or plasmids were cloned using the Gibson assembly (NEB E2611) cloning strategy (Gibson et al., 2009).

### Auxin-mediated degradation of mature GCN4 proteins

For degradation of mature 24xGCN4 proteins in embryos, L4 or young adult animals were transferred to NGM plates containing 1 mM auxin (IAA; Alfa Aesar) and seeded with OP50 *E. coli*, and were incubated overnight. Adult animals were splayed to collect embryos for microscopy. Auxin was used in experiments for Figs 1J,K, 5D, S2D, S3B-D and S3G,H.

### Heat-shock experiments

Heat-shock experiments were performed by transferring adult worms to pre-warmed M9. Animals were incubated at 20°C or 33°C for 30 min or at 37°C for 10 min and splayed immediately afterwards to obtain the embryos for microscopy.

### smiFISH

Custom smiFISH primary probes were designed against the *24xGCN4*, *tagBFP2* or *gfp* mRNA sequences using the Oligostan script in RStudio as previously described (Tsanov et al., 2016). A pool of 21-33 primary probes (IDT) was combined in IDTE buffer (pH 8.0) to a final concentration of 0.833 µM per probe. Primary probe sequences can be found in Table S3. Secondary (FLAP-Y) probes labeled with CAL Fluor 610 or Quasar670 were ordered from LGC Biosearch Technologies. Probes were annealed prior to each experiment by hybridizing 6 µl primary probes, 3 µl secondary probes, 3 µl NEB3 buffer and 19 µl H_2_O in a thermocycler at 85°C for 3 min, 65°C for 3 min and 25°C for 5 min. The smiFISH protocol was adapted from Parker et al. (2020). In brief, animals were collected from plates containing many gravid adults and washed three times in M9. Embryos were obtained by hypochlorite bleaching, washed three in M9 and transferred to 1.5 ml Eppendorf tubes. For fixation, embryos were resuspended in 1 ml acetone, submerged in liquid nitrogen for 1 min and incubated at −20°C for 10-60 min. Samples were treated with Stellaris Wash Buffer A (Biosearch Tech cat. SMF-WA1-60) containing 10% formamide (Invitrogen AM9342) for 5 min at room temperature and incubated with 100 µl Stellaris Hybridization Solution (Biosearch Tech, SMF-HB1-10) containing 0.91 µl hybridized smiFISH probes at 37°C for 30 min with shaking at 450 rpm in the dark. Embryos were washed with Stellaris Wash Buffer A containing 2 µg/ml DAPI at 37°C for 30 min with shaking at 450 rpm in the dark. Finally, samples were incubated with Wash Buffer B at room temperature for 5 min, mounted in Vectashield mounting medium (Vector laboratories) and coverslipped.

### smiFISH combined with immunostaining

Primary and secondary smiFISH probes were hybridized as described for smiFISH. The experimental protocol was adapted from Tocchini et al. (2021) and carried out in quadruplicate. Gravid adults were splayed on a poly-L-lysine-coated glass slides in 10 µl H_2_O. A coverslip was added and slides were transferred to liquid nitrogen. After a freeze crack, slides were immediately transferred to methanol at −20°C and incubated for 5 min. Slides were washed at room temperature in PBS (5 min), in PBS with 0.5% Tween-20 (10 min and 20 min) and in PBS (5 min). Samples were incubated in 50 µl fresh hybridization buffer [dextran sulfate (10% w/v) in one part formamide, one part 20×SSC and eight parts H_2_O] in a humidity chamber at 37°C for 1 h. Hybridization buffer was removed and 50 µl hybridization buffer containing 1 µl pre-hybridized probes and primary antibodies [GFP goat polyclonal (Abcam, ab6673) at 1/1000; RFP rabbit polyclonal (Rockland, 600-401-379) at 1/300] were added to the sample. Slides were incubated in a dark humidity chamber at 37°C for 6.5 h and at 4°C overnight. Samples were washed twice with wash buffer (one part formamide, one part 20×SSC and eight parts H_2_O) and incubated with 100 µl wash buffer containing secondary antibodies [Alexa Fluor 488 donkey anti-goat (ThermoFisher Scientific, A-11055) at 1/500; Alexa Fluor 555 donkey anti-rabbit (ThermoFisher Scientific, A-31572) 1/500] in a dark humidity chamber at 37°C for 1 h. Slides were washed twice with wash buffer, mounted with Vectashield mounting medium (Vector laboratories) containing 0.7 µg/ml DAPI (Sigma-Aldrich D9542) and coverslipped.

### Microscopy and image analysis

*C. elegans* larvae were paralyzed in a 10 mM tetramisole solution in M9 buffer (0.22 M KH_2_PO_4_, 0.42 M Na_2_HPO_4_, 0.85 M NaCl and 0.001 M MgSO_4_) and mounted on a 5% agarose pad. For imaging of embryos, adults were splayed in M9 to release the embryos before mounting on a 5% agarose pad. Wide-field imaging was performed using an Axioplan2 upright microscope (Zeiss), equipped with a DIC polarizer and an Axiocam MRm CCD monochrome camera (Zeiss) and Plan-Apochromat 63×/1.4 Oil DIC M27 objective. Confocal imaging for smiFISH was performed using a Nikon Ti-U microscope equipped with Plan Apo VC 100×/1.40 oil objective (Nikon, Japan) and Flash 4.0 v3 detector (Hamamatsu, Japan). Other confocal microscopy was performed using an Eclipse Ti2-E with perfect focus spinning disk (Nikon, Kōnan Japan) equipped with a CSU-X1-M1 confocal head (Yokogawa, Tokio Japan) and CFI Plan Apo λ 100×/1.45 oil objective. Images of the *erm-1^24xSunTag^* strain were acquired using an Andor iXon DU-885 camera. A Photometrics Evolve EMCCD camera was used for heat-shock experiments, smiFISH combined with immunostaining and timelapse imaging of SunTag reporter strains. For all other experiments, a Prime BSI sCMOS camera was used. For quantification, identical imaging approaches were applied within experiments. Microscopy data were acquired using Axiovision 4.x imaging software (wide-field microscopy), MetaMorph Microscopy Automation and image analysis software (spinning disk confocal) or Micromanager (re-scan confocal microscopy). Tracking of *erm-1* translation was performed using Imaris 10.0.1 (Oxford Instruments). All other image processing was performed in FIJI in a non-destructive manner (Schindelin et al., 2012). Areas outside the original field of view that became visible due to imaging rotation are indicated in white in the final figures.

### Quantification of localized translation

The distance of translation spots to the closest plasma membrane was calculated using Arivis Vision4D version 3.5.0. All analyzed embryos contained 40 to 60 cells and had a minimum of 25 translation spots. Embryonic cells were segmented based on the membrane marked by mCherry::PH and translation spots were detected by clustering of the scFv::GFP signal. The membrane signal was enhanced using a simple sharpening filter and Membrane Detection – Enhance Edges to ease segmentation of the individual cells. Detection of translation spots was improved using a discrete Gaussian denoising function to smooth out the signal and to filter out hot pixels. Embryonic cells were segmented using a Membrane-Based Segmenter and translation spots were segmented using a Blob Finder. The probability threshold of the blob finder operation was determined manually per embryo to achieve a correct segmentation of the translation spots. The split sensitivity of the blob finder operation was identical between *erm-1* and the randomized control but lower for the reporter to prevent over-segmentation. Everything outside the embryos of interest and scFv::GFP signal within cells that were clearly dividing was discarded. Finally, the distance between the center of each translation spot and the closest edge of the membrane was measured in three dimensions (3D) in µm and exported via an Excel file. The number of translation spots within 0-0.35 μm from the membrane was divided by the total number of translation spots to calculate the proportion of localized translation. The randomized control was generated by flipping only the scFv::GFP signal with respect to the *z*-axis to create a random distribution of translation spots while maintaining most translation spots within the embryo marked by the mCherry::PH. The exact same pipeline was followed in Arivis as the original data file to prevent computational bias.

### Quantification of overlap between translation spots and mRNA

To quantify the overlap between translation spots and mRNA, *z*-stacks of embryos – stained for mRNA and protein (combined smiFISH and immunofluorescence protocol) – were acquired at 0.4 μm intervals. Images were included for analysis if both mRNA and protein staining were homogeneous throughout the embryo. Maximum intensity projections of six *z*-stacks were generated using FIJI and background signal outside of embryos was discarded (Schindelin et al., 2012). FIJI ComDet v.0.5.5 was used to detect mRNA and translation spots in an unbiased manner, and to calculate the integrated intensity of each spot (Katrukha, 2020). Only GFP spots with an integrated intensity >25,000 were classified as translation spots and included in the analysis. The percentage of overlap was calculated by dividing the number of translation spots that overlap with mRNA by the total number of translation spots. Only embryos with more than three translation spots were used for analysis.

### Quantification of translation spot numbers, intensity and re-localization efficiency

Translation spots were analyzed in 30-60 cell stage embryos of *erm-1^24xSunTag;20xPP7^*, *erm-1^24xSunTag;8xPP7^* and *erm-1^24xSunTag;2xPP7^* with or without re-localization by PCP::mCherry::NPP-9 as well as SunTag reporter strains. Animals expressing the AID system were cultured overnight on NGM plates containing 1 mM auxin (IAA; Alfa Aesar) and seeded with OP50 *E. coli*. Embryos were collected from plates and imaged with 0.4 μm intervals over the *z*-axis. FIJI was used to generate maximum intensity projections of five *z*-stacks and to outline the embryos. Unbiased translation spot detection was performed using the FIJI plug-in ComDet v.0.5.5 (Katrukha, 2020). For *erm-1^24xSunTag;20xPP7^*, *erm-1^24xSunTag;8xPP7^* and *erm-1^24xSunTag;2xPP7^* embryos without or in combination with expression of PCP::mCherry::NPP-9, only GFP spots with an integrated intensity of more than 2000 were classified as translation spots and included in the analysis, as dimmer spots likely represent mature proteins rather than active translation sites. No intensity threshold was used for SunTag reporter strains, since as all contained the AID system, ensuring mature protein degradation. Translation spots were visually categorized as ‘Nuclear pore’, ‘Plasma membrane’ or ‘Cytosol’. Small differences in localization may be missed due to lower intensities of membranes orientated parallel to the *z*-axis or the absence of a nuclear marker in control strains. Mitotic cells were excluded from analysis as background signal hindered analysis and spots could not be categorized as ‘close to the nuclear pore’. To calculate the percentage of localized translation spots, the number of spots in each category was divided by the total number of spots per embryo. Embryos were included in the re-localization efficiency analysis if they contained more than seven translation spots. All embryos were included for analysis of translation spot number and intensity.

### Quantification of membrane and cytoplasmic ERM-1 levels in four-cell stage embryos

Embryos of *erm-1::GFP* and *erm-1::1xGCN4; scFv::GFP* strains were imaged and analyzed using FIJI (Schindelin et al., 2012). Apical ERM-1 levels were obtained from the average of the peak intensity of three line scans perpendicular to the membrane between the ABa and ABp cells. Cytoplasmic ERM-1 levels were calculated from the average of the mean intensity of three cytoplasmic regions in the ABp cell. Apical ERM-1 levels were divided by cytoplasmic ERM-1 levels to calculate the ratio.

### Quantification of ERM-1::GFP levels and apical/cytoplasmic ratios in 1.5 fold stage embryos and larvae

ERM-1::GFP levels were analyzed in 1.5-fold stage embryos and L1 larvae using FIJI (Schindelin et al., 2012). To obtain *erm-1::GFP/+* embryos and larvae, *erm-1::GFP* animals were crossed and heterozygous offspring was selected. L1 larvae were collected from plates and included in the analysis if they contained two primordial germ cells to ensure developmental synchronization. Fluorescence intensity of ERM-1::GFP was measured using the average of the peak histogram intensity from three line scans (30 pixels wide) perpendicular to the apical membrane of 2R, 2L, 3R, 3L, 4R and 4L intestinal cells in embryos, or 2R, 2L, 3R and 3L intestinal cells in larvae. Background fluorescence was measured in three regions outside embryos or larvae, averaged and subtracted from the apical ERM-1::GFP levels. For the apical/cytoplasm ratio in 1.5-fold stage embryos, cytoplasmic ERM-1::GFP intensity was averaged from three regions in the cytoplasm of 2R, 3R and 4R intestinal cells. After background subtraction, apical ERM-1::GFP levels were divided by cytoplasmic ERM-1::GFP levels to calculate the ratio.

### Fluorescent recovery after photobleaching of ERM-1::GFP

L1 larvae expressing *erm-1::GFP^8xPP7^* or *erm-1::GFP^20xPP7^* with or without re-localization by PCP::mCherry::NPP-9 were collected from plates and analyzed if they contained two primordial germ cells, ensuring developmental synchronization. Photobleaching was performed using a consistent laser setting for all animals and resulted in a photobleached area of ∼90 pixels in width. Selected areas were never completely photobleached to ease analysis. Timelapse videos were recorded prior, during and immediately after photobleaching. Recovery was followed at 20 s intervals for 20 min. Each time-lapse frame was analyzed in FIJI (Schindelin et al., 2012). The ERM-1::GFP intensity in the bleached region was measured using the peak histogram intensity of a 20 pixel wide line scan perpendicular to the apical intestinal membrane. Background fluorescence was subtracted using the average intensity of a region outside the animal. Measurements of each time-lapse frame were corrected for acquisition photobleaching, which was determined from the background-subtracted peak histogram intensity of a non-photobleached apical intestinal region, normalized to the corresponding intensity of five pre-photobleaching frames. The corrected ERM-1::GFP intensity within the bleached region of the first time-lapse frame after photobleaching (t=0) was subtracted from all corrected intensities to establish as zero baseline. Fluorescent recovery after photobleaching (FRAP) was calculated from the change in corrected intensity, normalized against pre-photobleaching values. For each animal, two FRAP measurements were averaged.

### Quantification of ACT-5 enrichment

Apical enrichment of YFP::ACT-5 was determined in FIJI by dividing the apical levels by the cytoplasmic levels because of variable expression levels of the transgene. Analysis was performed in intestinal cells of comma to 1.5-fold embryos. Apical protein levels were calculated by averaging the peak histogram intensity values of three 15-pixel wide line scans perpendicular to the apical membrane of the 2R, 2L, 3R and 3L intestinal cells of each animal. Cytoplasmic protein levels were obtained from the average of the mean intensity of three regions in the cytoplasm of the 2R and 3R intestinal cells per animal. Background levels were measured in three regions outside the embryos, averaged, and subtracted from the apical and cytoplasmic protein levels.

### Estimation of smiFISH probe binding efficiency

Probe binding efficiency for smiFISH was estimated using two different primary probes (against *24xGCN4* and *bfp*) targeting the same *24xGCN4::T2A::BFP::h2b* reporter mRNA, hybridized with different secondary probes (Quasar670 and CAL Fluor 610, respectively). The protocol for smiFISH and immunostaining was followed as described above. mRNA spots were detected using ComDet v.0.5.5 in FIJI (Katrukha, 2020; Schindelin et al., 2012). Fluorescent spots with an integrated intensity greater than 50,000 were classified as mRNA spots and included in the analysis. Data were exported to MS Excel to calculate the number of mRNA spots that overlapped between different channels. The number of overlapping spots was divided by the total number of mRNA spots for each channel to calculate the probe-binding efficiency.

### Brood size

The progeny size of wild-type, *erm-1^24xSunTag^* and *erm-1^24xSunTag;20xPP7^* strains with or without re-localization by PCP::mCherry::NPP-9 was determined over the course of 72 h from egg-laying onset. Ten healthy looking animals were cultured at 20°C for every 24 h before all laid eggs and larvae were counted and parental animals were transferred to fresh plates.

### Quantification of larval body length

To collect synchronized L1 larvae, embryos were obtained by hypochlorite bleaching and hatched overnight in M9. Larvae were filtered through a 20 µm nylon net filter (Merck NY2004700) to discard unhatched eggs, and incubated at 20°C for 72 h. At 0 h (before incubation) and after 24 h of incubation, animals were imaged using an Axioplan2 wide-field microscope (Zeiss). At 48 and 72 h of incubation, animals were imaged using a S9i Digital Stereo Microscope (Leica) at 4× magnification. Images were analyzed in FIJI to calculate the body length as a measurement of growth (Schindelin et al., 2012). Animals were manually outlined and the plug-in Analyze Skeleton (2D/3D) (Arganda-Carreras et al., 2010) was used to determine the length of the worm. L1 larvae were omitted from the analysis if animals on the same plate had started egg laying.

### Quantification of intestinal defects in embryos

To quantify the number of intestinal abnormalities, we analyzed the intestinal morphology in 2.5- and 3-fold embryos. Embryos were collected from plates and imaged to assess lumen structure. Intestinal abnormalities were scored based on the presence of luminal twists, constrictions or cysts.

### Larval morphology assessment

To quantify intestinal defects in larvae, intestinal morphology was examined based on DIC images and scored as ‘no defect’ (no irregularities), ‘mild defects’ (little widening of the lumen), ‘medium defect’ (larger widening of the lumen or small constrictions) or ‘severe defects’ (severe widening of the lumen or significant constrictions). Larvae were synchronized by hypochlorite bleaching of gravid adults and overnight hatching of collected embryos in M9. Morphological phenotypes of *erm-1^24xSunTag;20xPP7^* with or without re-localization by PCP::mCherry::NPP-9 were characterized by E.v.d.S. and at least three others, after which a consensus grade for each image was determined. Samples were anonymized. If no consensus was reached (e.g. in case of a tie), the grade assigned by E.v.d.S was used (8/90 final grades). Morphological phenotypes of *erm-1^24xSunTag;8xPP7^* with or without re-localization by PCP::mCherry::NPP-9 were characterized by E.v.d.S.

### Statistical analysis

Statistical analyses were performed using GraphPad Prism v.10. A D'Agostino-Pearson test was used to assess normality. For normally distributed data, comparisons of two populations were made using an unpaired Student's *t*-test (with Welch's correction if the standard deviations of the populations differed significantly), and comparisons of more than two populations were carried out using a one-way ANOVA followed by a Bonferroni's multiple comparison test. For data not drawn from a normal distribution, comparisons of two populations were made using a Mann–Whitney test, and comparison of more than two populations was performed using a Kruskal–Wallis test followed by a Dunn's multiple comparison test. For FRAP data, a repeated-measurement two-way ANOVA with Geisser-Greenhouse correction was used to compare the different populations. This statistical teste assesses differences in recovery dynamics over time by considering all time points simultaneously, thereby evaluating overall trends rather than individual time points. The used statistical tests and sample sizes are described in the figure legends. Sample sizes were not pre-determined by statistical tests. No data were excluded from analysis, unless described in the Materials and Methods.

### Declaration of AI-assisted technologies in the writing process

During the preparation of this work, the authors used an AI language model (ChatGPT, provided by OpenAI) to improve language and readability. After using this tool, the authors reviewed and edited the content as needed, and take full responsibility for the content of the publication.

## Supplementary Material



10.1242/develop.204435_sup1 Supplementary information

Table S1. Overview of strains used in this study This table lists the strains used in this study, detailing their generation methods and the figures in which the corresponding data is presented. Since data are too large to fit in PDF, Table S1 is provided in a separate excel file.

Table S2. Overview of knock-ins generated in this study This table details the knock-ins generated in this study, including the guide RNAs and repair templates used for their creation. Since data are too large to fit in PDF, Table S2 is provided in a separate excel file.

Table S3. Primary probes used for smiFISH, related to Methods This table lists the primary smiFISH probes used in this study. Table S3 is provided in a separate excel file.
