# Supplementary material for: Measuring and manipulating localized translation of erm-1 in the C. elegans embryo
Source: Development. 2025 May 19;152(10):dev204435. doi: 10.1242/dev.204435 (PMC12148021; doi:10.1242/dev.204435)
Supplement: Supplementary information [file develop-152-204435-s1.pdf]

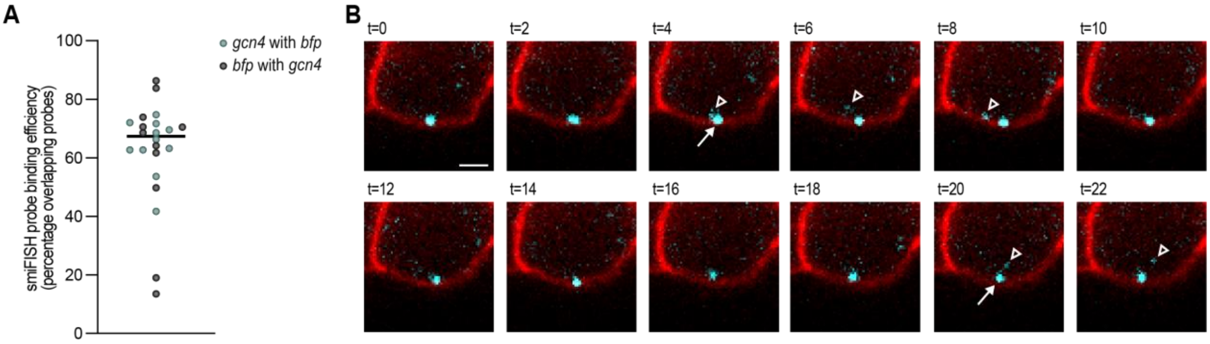

Fig. S1. smiFISH efficiency and release of GCN4 proteins from translation spots

(A) smiFISH probe binding efficiency as measured by the overlap in *24xGCN4* and *BFP* smiFISH signals on *24xGCN4::t2a::BFP::h2b* mRNAs. Green dots represent the percentage *24xGCN4* smiFISH spots overlapping with *BFP* smiFISH spots, grey dots represent the percentage of *BFP* smiFISH spots overlapping with *24xGCN4* smiFISH spots, per embryo (n=11 embryos). **(B)** Time-lapse imaging of the release of mature protein from a translation spot over a 22 second period in an embryo expressing the SunTag antibody (*scFv::GFP*, in cyan), a translation imaging reporter (*eft-3p::24xGCN4::AID::T2A::BFP::h2b::20xPP7::tbb-2 3'UTR*), the membrane marker (mCherry::PH; depicted in red) and the PCP protein (PCP::mCherry::PH; also depicted in red). The translation spot remained visible throughout the live imaging and is indicated by arrows. GCN4 proteins released from the translation site were transiently visible and are indicated by open triangles. Scale bar: 1 μm.

Development • Supplementary information

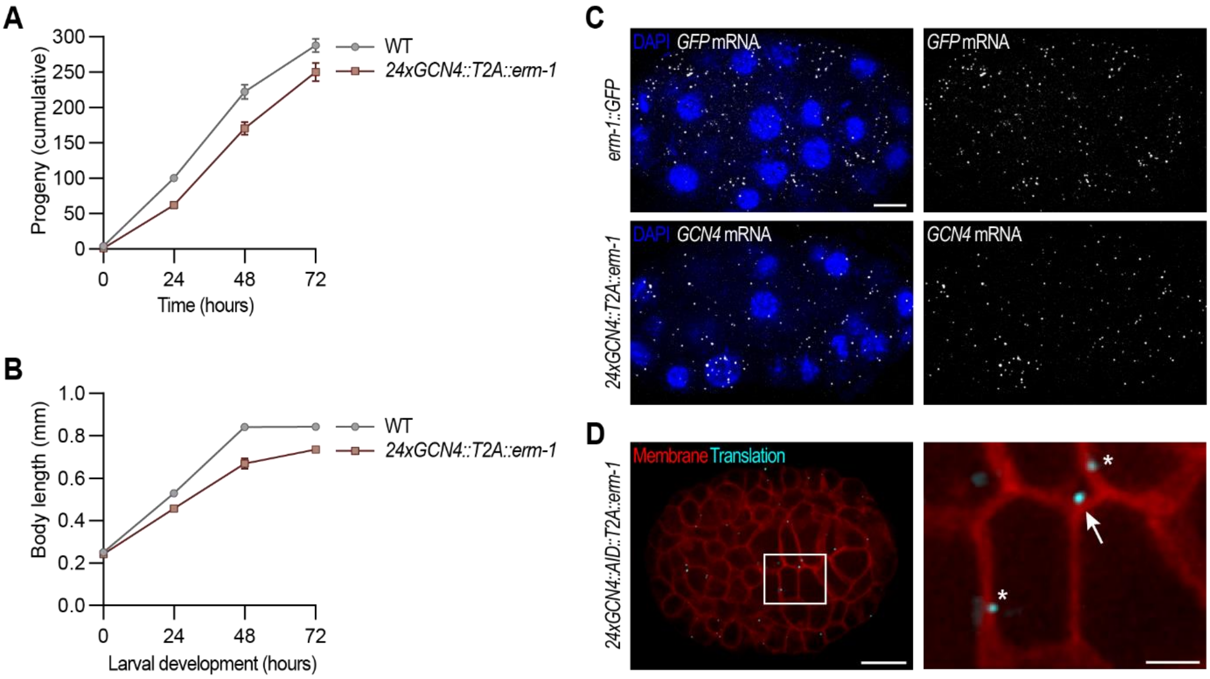

Fig. S2. Characterization of the *erm-1*^{24xSunTag} strain

(A-B) Quantification of brood size (A) and larval growth (B) of WT and *erm-1*^{24xSunTag} strains. n=10 animals of both strains in (A). For (B): n=23, 35 for t=0; n=29, 32 for t=24; n=17, 24 for t=48; n=15, 12 for t=72; for WT and *erm-1*^{24xSunTag}, respectively. **(C)** Representative images showing similar *erm-1* mRNA (smiFISH signal; gray) levels in *erm-1::GFP* embryos (upper images) or *erm-1*^{24xSunTag} embryos. Nuclei are indicated by DAPI (blue). Scale bar: 10 µm. **(D)** Translation of *erm-1* (cyan) in close proximity of the basolateral (asterisks) and apical (arrow) membranes (red) of developing intestinal cells in a *24xGCN4::AID::T2A::erm-1* embryo. Scale bar: 10 µm (embryo); 2.5 µm (zoom-in).

Development • Supplementary information

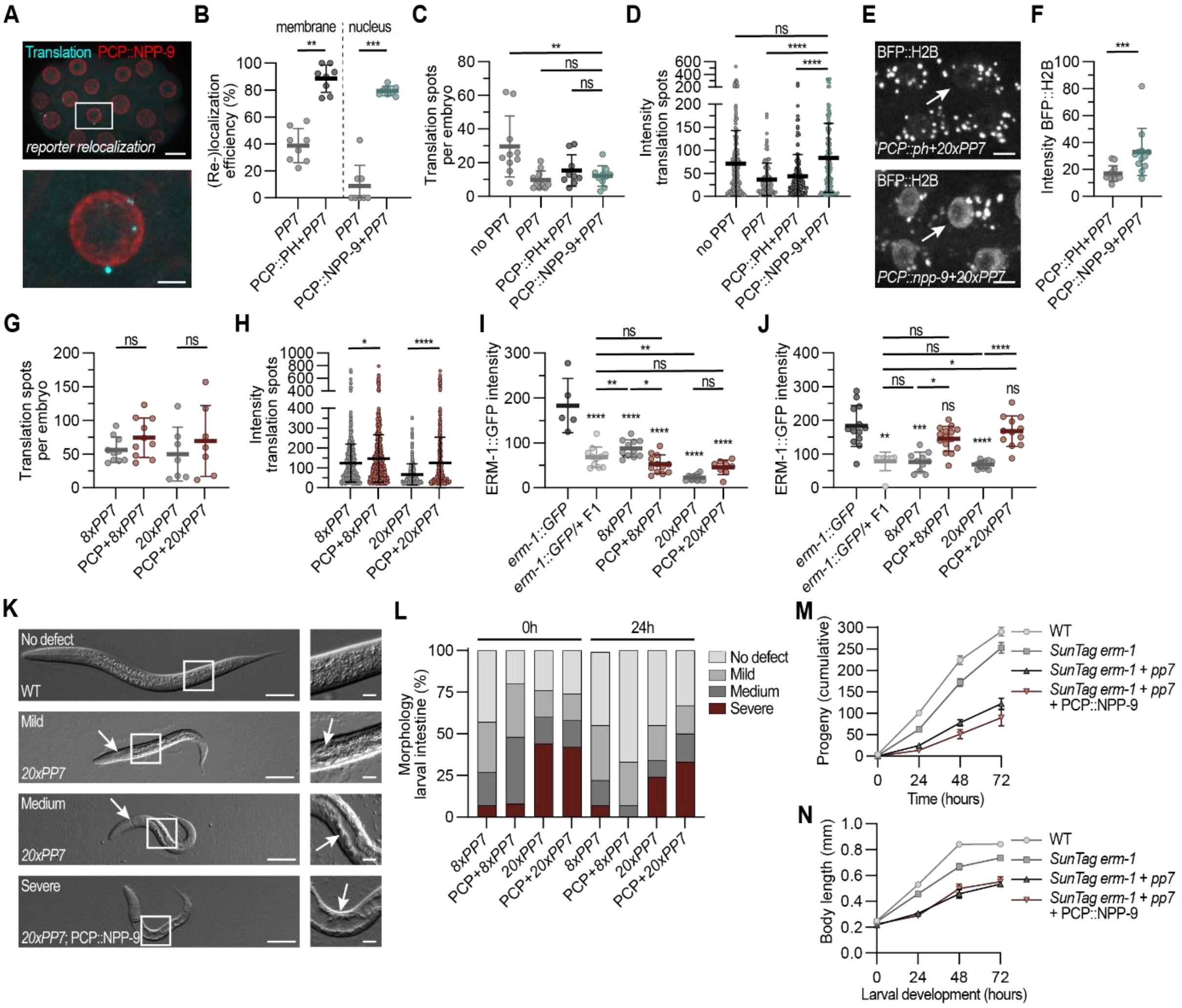

Fig. S3. The nuclear pore is a favorable cellular location for translation and re-localization of *erm-1* to the nuclear pore affects ERM-1

(A) Representative embryo in which reporter mRNAs (*24xGCN4::AID::T2A::BFP::h2b::20xPP7::tbb-2 3'UTR*) are re-localized to the nuclear pore via PCP::mCherry::NPP-9; (red). Translation spots in cyan. Scale bar: 10 µm (embryo); 2.5 µm (zoom-in). **(B)** Quantification of translation (re-)localization efficiency to the membrane or nuclear pores of reporter mRNAs with or without PCP::mCherry::PH or PCP::mCherry::NPP-9 expression (n=8 per strain). Errors bars indicate mean ± SD. Test of significance: Kruskal-Wallis test with Dunn's multiple comparisons test. ns = not significant, **p≤0.01, ***p≤0.001. **(C)** Quantification of the number of translation spots per embryo in SunTag reporter strains with or without 20xPP7 and with re-localization to the membrane or nuclear pore (n=10, 11, 9, 10). Errors bars indicate mean ± SD. Test of significance: One-way ANOVA with a Bonferroni's multiple comparison test. ns = not significant, **p≤0.01. **(D)** Quantification of translation spot intensities of reporter mRNAs with or without 20xPP7 and with re-localization to the membrane or nuclear pore. Each dot represents a single translation spot (n=296, 107, 138, 121). Errors bars indicate mean ± SD. Test of significance: Kruskal-Wallis test with Dunn's multiple comparisons test. ns = not significant, ****p≤0.0001. **(E)** Representative images showing BFP::H2B levels in SunTag reporter strains with re-localization to the membrane (upper panel) or nuclear pore (lower panel). Scale bar: 5 µm. **(F)**

Quantification of BFP::H2B levels in SunTag reporter strains with re-localization to the membrane or nuclear pore (n=12 for both strains). Errors bars indicate mean ± SD. Test of significance: Two-tailed Mann Whitney test, ***p≤0.001. **(G)** Quantification of the number of translation spots per embryo, with or without *erm-1* re-localization (n=10, 10, 7, 7). Errors bars indicate mean ± SD. Test of significance: Kruskal-Wallis test with Dunn's multiple comparisons test. ns = not significant. **(H)** Quantification of translation spot intensities in embryos with or without erm-1 re-localization. Each dot represents a single translation spot (n=564, 745, 350, 487). Test of significance: Kruskal-Wallis test with Dunn's multiple comparisons test, *p≤0.05, ****p≤0.0001. **(I-J)** Quantification of ERM-1::GFP levels in intestinal cells of embryos (I) or larvae (J) in *erm-1::GFP*, *erm-1::GFP/+* and *erm-1::GFP*^{PP7} strains with or without *erm-1* re-localization (n=5, 11, 11, 12, 9, 8 for (I); n=13, 9, 10, 15, 11, 12 for (J)). Test of significance: One-way ANOVA with Bonferroni's multiple comparisons test for (I); Kruskal-Wallis test with Dunn's multiple comparisons test for (J). ns = not significant, *p≤0.05, **p≤0.01, ***p≤0.001, ****p≤0.0001. **(K)** Representative DIC images, showing the larval defects observed in the indicated genotypes. Insets at the right. Arrows: morphological defects in the intestine. Scale bar: 50 µm (larvae); 10 µm (zoom-in). **(L)** Quantification of morphological defects in larvae with or without *erm-1* re-localization, at 0 and 24 hours after hatching (n=30, 25, 25, 19, 27, 27, 29, 18). **(M-N)** Quantification of brood size (M) and larval growth (N) of WT strains, *erm-1*^{24xSunTag} strains and *erm-1*$^{24xSunTag;20xPP7}$ strains with or without PCP::mCherry::NPP-9 expression. n=10 for each condition in (M); n=23, 35, 25, 19 for t=0; n=29, 32, 22, 19 for t=24; n=17, 24, 24, 20 for t=48; n=15, 12, 28, 24 for t=72; WT, *erm-1*^{24xSunTag}, *erm-1*$^{24xSunTag;20xPP7}$ with or without PCP::mCherry::NPP-9 expression, respectively in (N). Data for figures (M) and (N) have been re-used from Figures S2A,B.

Development • Supplementary information

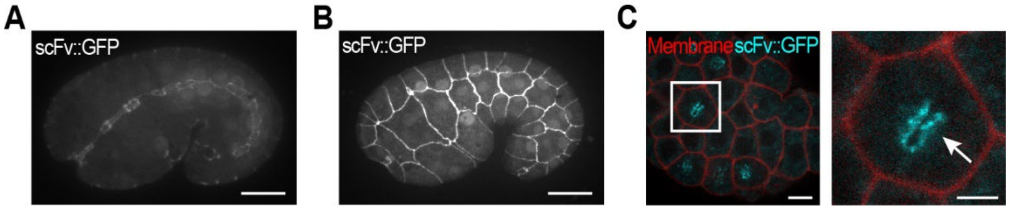

Fig. S4. Affinity of scFv::GFP proteins and GCN4 peptides to cellular structures

(A-B) Representative images of an scFv::GFP expressing embryo, with scFv::GFP localization to structures close to epithelial apical junctions. Scale bar: 10 µm. **(C)** Affinity of GCN4 peptides to structures near the DNA of mitotic cells, in an *erm-1*^{24xSunTag} embryo. Inset at the right. Arrow: GCN4 peptides labeled by scFv::GFP near the DNA of an mitotic cell. Scale bar: 10 µm (larvae); 5 µm (zoom-in).

Table S1. Overview of strains used in this study

This table lists the strains used in this study, detailing their generation methods and the figures in which the corresponding data is presented. Since data are too large to fit in PDF, Table S1 is provided in a separate excel file.

Available for download at
https://journals.biologists.com/dev/article-lookup/doi/10.1242/dev.204435#supplementary-data

Table S2. Overview of knock-ins generated in this study

This table details the knock-ins generated in this study, including the guide RNAs and repair templates used for their creation. Since data are too large to fit in PDF, Table S2 is provided in a separate excel file.

Available for download at
https://journals.biologists.com/dev/article-lookup/doi/10.1242/dev.204435#supplementary-data

Table S3. Primary probes used for smiFISH, related to Methods This table lists the primary smiFISH probes used in this study. Table S3 is provided in a separate excel file.

Available for download at
https://journals.biologists.com/dev/article-lookup/doi/10.1242/dev.204435#supplementary-data

Development • Supplementary information

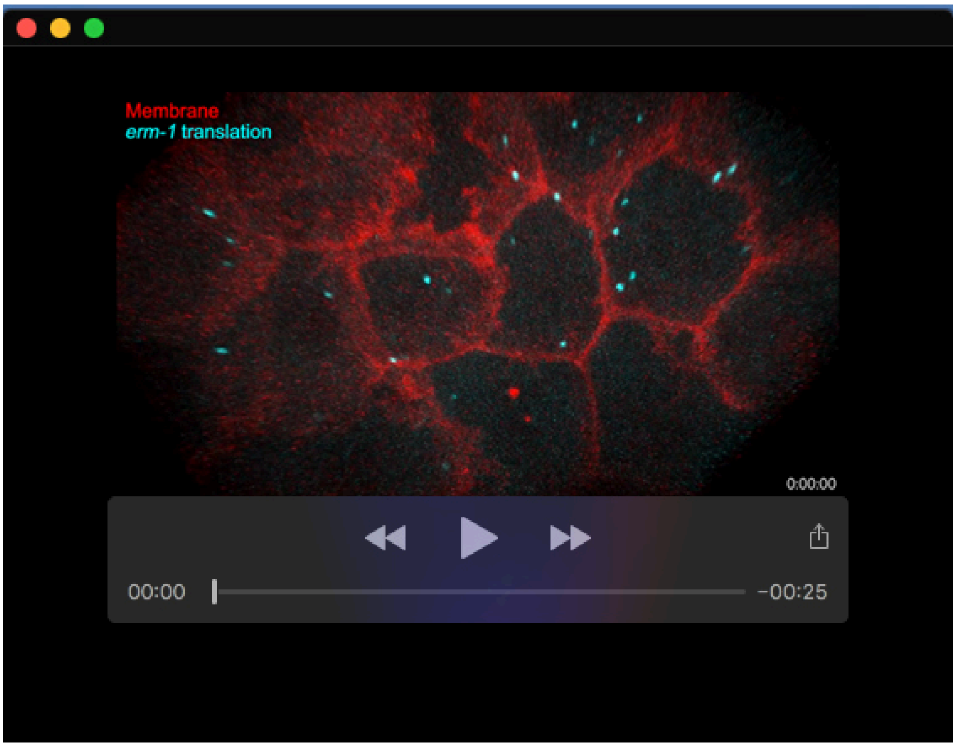

Movie 1. Movie of erm-1 translation, related to Fig. 4.

Timelapse movie of data from Figure 4. First, erm-1 translation moves from the cytoplasm to the plasma membrane where it remains. Second, an erm-1 translation spot dynamically interacts with the plasma membrane.

Development • Supplementary information
