# Supplementary material for: Measuring and manipulating localized translation of erm-1 in the C. elegans embryo
Source: Development. 2025 May 19;152(10):dev204435. doi: 10.1242/dev.204435 (PMC12148021; doi:10.1242/dev.204435)
Supplement: Supplementary file 1 [file develop-152-204435_review_history.pdf]

Measuring and manipulating localized translation of *erm-1* in the *C. elegans* embryo

Elise van der Salm, Esther Koelewijn, Mette Schroeder, Erica van der Maas, Olga Jarosińska, Max Eeken and Suzan Ruijtenberg
DOI: 10.1242/dev.204435

Editor: Swathi Arur

Review timeline
Original submission: 5 October 2024
Editorial decision: 2 November 2024
First revision received: 18 March 2025
Editorial decision: 4 April 2025
Second revision received: 10 April 2025
Accepted: 11 April 2025

Original submission

First decision letter

MS ID#: dev.204435

MS TITLE: Measuring and manipulating localized translation of erm-1 in the C. elegans embryo

AUTHORS: Elise van der Salm; Esther Koelewijn; Mette Schroeder; Erica van der Maas; Olga Jarosińska; Max Eeken; Suzan Ruijtenberg

Dear Dr Ruijtenberg,

I have now received all the referees' reports on the above manuscript, and have reached a decision. The referees' comments are appended below, or you can access them online: please go to:

As you will see, the referees express considerable interest in your work, but have significant recommendations to improve the rigor, claims, and clarity of the study. In general, both reviewer 1 and 3 ask for functional significance of the localized erm-1 translation, which is one of the major claims of the study. All three reviewers provide excellent suggestions for both improving the readability of the paper, as well as for providing data that matches the conclusions. If you are able to revise the manuscript along the lines suggested, which involve further experiments, I will be happy receive a revised version of the manuscript. Your revised paper will be re-reviewed by one or more of the original referees. Please also note that Development will normally permit only one round of major revision.

Reviewer 1

Advance summary and potential significance to field

The manuscript by van der Salm et al reports on the adaptation of the SunTag system for tracking co-translational targeting in C. elegans and apply this technique to understand the mechanisms and role of co-translational targeting and localized translation of the protein ERM-1.

© 2025. Published by The Company of Biologists under the terms of the Creative Commons Attribution License (https://creativecommons.org/licenses/by/4.0/).

As a general resource, it should be noted that the general principles of the system, including AID degradation have been developed in other systems in prior work. That said, the adaptation and optimisation of the system and demonstration of proof of principle in worms make it a potentially valuable resource for the community. I have a few minor comments in this regard and in general find the validation of the tool well supported.

Regarding ERM-1, the report provides a nice example of how the use of live imaging approaches enabled by the SunTag provide a much richer and dynamic picture of erm-1 translation. erm-1 mRNA has previously been reported to colocalize with ERM-1 protein in C. elegans in a translation-dependent manner, suggesting a model in which the emergence of the N-terminal membrane binding FERM domain targets mRNA to the membrane, enabling localised translation. Using the SunTag system, the authors can demonstrate local translation and additionally report on heterogeneity and dynamics of localisation/translation. For example, their data suggest that translation is enriched at membranes, but is not apically enriched, suggesting that apical enrichment occurs post-translationally. The heterogeneity such as switching between translated and non-translated states as well as between membrane and cytoplasmic states also raises interesting questions. Finally, the authors report defects in ERM-1 function (though not localisation) when they mislocalize mRNA (and thus translation) to the nuclear envelope, suggesting a link between translation localisation and function. However, as I note below I am less convinced of this final point.

Finally, I appreciate the extensive discussion on potential pitfalls and caveats in applying this approach so that readers have a good grasp of the pros/cons/limits of the system. This is particularly useful for a resource article.

Comments for the author

Major comments

My primary concern relates to claims that localised translation of erm-1 is important for function. This conclusion relies on the use of an mRNA tethering strategy based on the hairpin / hairpin binding protein PP7/PCP which is used to mislocalize erm-1 mRNA to the nuclear envelope. Complicating the interpretation of these experiments is that the SunTag and PP7 tagging already induce some defects on their own independent of targeting. The authors acknowledge this issue and show some specific ERM-1 associated defects, but interpretations are nonetheless complicated. Some particular issues:

First, PP7/PCP (mistargeted) and PP7(tag controls) both show similar phenotypes, though the severity appears somewhat worse for PP7/PCP vs PP7 controls. In the case of Figure 5E, the frequency of defects is similar, though the degree of severity is significantly different. In the case of Figure 5G, it is unclear if the observed differences are significant (see comment below on numerical data) and even so are relatively minor.

Second, both PP7 and PP7/PCP appear to show reductions in ERM expression suggesting the PP7 hairpins and potentially PCP binding to hairpins influence expression. Indeed, it appears that levels are lower in the PP7/PCP compared to PP7 alone, though quantification is noticeably lacking here.

Given these points, it is difficult to interpret the impact of targeting on phenotypes. Specifically:

- Are phenotypes simply due to expression differences?

- Is the phenotype due to mistargeting or simply to the PP7/PCP tethering per se?

- if reduced expression is specific to nuclear mistargeting, is this due to the lack of membrane targeting or more to the nature of the mistargeting, for example if the nuclear pore environment is problematic? The latter would be less interesting as it is unlikely to end up there?

For the first point, more accurate quantification of expression differences, partial RNAi / heterozygotes, or overexpression would be informative here.

© 2025. Published by The Company of Biologists under the terms of the Creative Commons Attribution License (https://creativecommons.org/licenses/by/4.0/).

For the latter two points, it would be informative to compare the effects of distinct PCP-tethers, e.g. free PCP, plasma membrane, mitochondrial, nuclear. If plasma membrane targeting were critical for function, I would expect that free PCP and plasma membrane binding PCP should not enhance the phenotype of PP7 alone. In an ideal world, one would be able to disrupt the endogenous targeting signal (the FERM domain) and rescue via membrane PCP tethering, but as the FERM domain is critical for function, it is unclear how to approach this problem. Perhaps adding an mis-targeting domain to the SunTag sequence before the T2A to compete with the FERM domain. That said, the use if different PCP-tethers may be sufficient.

Additional minor comments

I am not an expert in this area, but the SunTag is built around the GCN4 leucine zipper. Does it show intrinsic di-/oligomerisation and would this impact interpretation?

I may be misunderstanding something, but in Figure 2 and associated text, it could be clearer why the membrane targeting of mRNA via the PP7/PCP system was important for demonstrating coincidence of mRNA and the SunTag reporter. Presumably one could look at colocalization of mRNA and the SunTag without this. I presume it aided tracking, but was unclear.

Line 188: "We observed a slightly slower growth rate..." Does this refer to Figure S3E?

Figure S2B, Line 197: Cites translation being enriched in intestinal precursors. This is not critical for the paper, but given the fact that only one embryo is shown, there are relatively few spots, and no quantification, I don't think the authors can draw a conclusion here.

Figure 5E, 5G: It would be helpful for readers to have access to the underlying numerical data for these frequency plots to judge the significance of the relevant differences. Particularly in the case of Figure 5G, could the differences be expected based on chance alone? How robust are the frequencies across replicates? Note for completeness, Figure S3B also lacks underlying numerical data, but the differences here are so large I am less worried.

Figure S3C (Differences in expression): Quantification missing.

<u>Reviewer 2</u>

This manuscript develops a novel nascent peptide imaging system that can be used to assess translation dynamics in real time and at the single-molecule level in C. elegans. The authors apply their technology to the study of ERM-1, a conserved membrane-linker protein crucial for cell function and development. Previous work had shown the erm-1 mRNA and protein both co-localize at the plasma membrane in a translation-dependent manner. However, the dynamics of the process and their functional relevance could not be assessed using previous methods.

Here, the authors leverage innovative live imaging approaches to address both questions. Using an adapted SunTag system, they discover ERM-1 translation does indeed occur at the membrane and that its behavior is strikingly dynamic and heterogenous. The group next generated a method to artificially tether the erm-1 mRNA to an ectopic locale in the cell. This resulted in developmental defects that were specifically associated with the ectopic tethering. Therefore, they verified that the site where ERM-1 is translated in the cell impacts its function.

This paper makes several significant and novel contributions to our understanding. #1) It develops translational imaging technology in C. elegans, a breakthrough feat. #2) It introduces an ectopic mRNA tethering approach in C. elegans that has the potential to be adapted for other transcripts. #3) It illustrates that ERM-1 is locally translated as hypothesized with some surprising behaviors. #4) It illustrates that ERM-1's local translation is required for its function.

The value of this last contribution cannot be understated. Multiple mRNAs in C. elegans have been shown to have translation-dependent mRNA localization. This is interesting but the functional relevance was heretofore unestablished. Frustratingly, protein motifs required for localization are typically also crucial for function, preventing of ablation of one without the other. The PP7

© 2025. Published by The Company of Biologists under the terms of the Creative Commons Attribution License (https://creativecommons.org/licenses/by/4.0/).

approach adapted here circumvents this technical limitation and can be used in the study of other transcripts.

Other highlights of the paper:
* The authors find that erm-1 mRNA may release from the membrane after completion of one round of translation and then re-associate at the next round. This is an unexpecting finding that underscores the value of a live imaging approach.
* The paper quantifies how PP7 stem loops can lead to disrupted mRNA behavior
* There are several elegant controls utilized
* The shortcomings and limitations are clearly described

Overall, I have only one Major Concern, a few Mid-Level Concerns, and a list of completely optional Minor Suggestions and General Curiosities. All are listed below.

In sum, I recommend this paper for publication in Development with minor revisions.

Major Concern
* My major concern may be a limitation of the submission process. The paper needs movies to illustrate the dynamical movements of ERM-1. Please include a selection of movies for the readers to evaluate.

Mid-level concerns
* What is the logic that fully translated proteins are dimmer than the actively translated protein? Is the idea that they are undergoing auxin-mediated decay? If so, do they disappear? Or do they remain dim?
* Line151. Consider rewording to avoid the word "termination" here as it implies translational termination. What about "translational repression", "stress", or "heat stress" - terms that more accurately reflects the process.
* Figure 2B. Why was this particular statistical test chosen? Maybe include a statement like, "…to account for differing numbers of replicates" to explain the departure from the Welch's t-test that was used in the other parts of the paper.
* Figure 2D. Can you clarify the Y-axis either in the figure or legend or both? The way it is written as "overlap", it is unclear whether this is the percent of translation spots that contain mRNA or the percentage of mRNA spots that contain translation. Something like "Overlap spots as a fraction of the total translation spots".
* Can you clarify the idea that there is germline silencing happening in early embryos? Or, is the eft-3 promoter not transcribed until the 16-cell stage? If the reporter is silenced in early embryos, how are the 2-cell and 4-cell images in Fig 1C - F achieved?
* Page 5. Line 158 - 164. The authors state that translation spots that do not have a corresponding mRNA probe spot must indicate that the smiFISH technology has low labeling efficiency. Low labeling efficiency may play a role, but how does one distinguish low labeling efficiency from a fully translated peptide, one that has dissociated from the mRNA but has not yet degraded?
* How large is the GCN4 protein/peptide? Is this a protein from yeast? Or is this a shortened peptide? Some indication of the length and sequence would be helpful to envision the size of the N-terminal tag.
* On that note, wild-type ERM-1 requires a functional N-terminal FERM domain for localization. Because the GCN4 tags are placed N-terminal to the FERM tag, it is possible that any time lapse or pauses in translation between the GCN4 translation and FERM domain may lead to a delay in localization to the membrane. This may account for some fraction of the cytoplasmic translating mRNAs. Specifically, mention this caveat on Pg. 7 Lines 231 - 232.
* How does the addition of GCN4 and/or PP7 sequences impact mRNA abundance?

Minor Points
* Fig 1D,E are referenced in opposite order in the figure and in the text.
* Figure 1 Legend. 1K. The Figure legend for 1K references two figures, but only one is shown
* Figure 2D figure legend. Line 702. "where" should be "were"'
* Page 7. Line 237. Missing period after the word ERM-1.
* Figure 4A. Could you label the dotted rectangles that indicate insets as either "B" or "C"? It will aid the reader in

© 2025. Published by The Company of Biologists under the terms of the Creative Commons Attribution License (https://creativecommons.org/licenses/by/4.0/).

* Page 8. Line 262. In this section, could you highlight that there are no other copies of erm-1 in the strain?
* Page 12. Line 406 - Again, how big is each GCN4 unit? Also, this explanation doesn't account for any pauses in translation that may occur
* Figure 3C. What is randomized here? The GCN sequence? Or was it a random, computational selection of X, Y, Z coordinates?
* Figure 5C. can you clarify the Z-stacking here? Is it single slice, a few slices, or max project?
* Methods: Progeny size. Should this be "Brood Size"? Progeny size implies a measurement of worm dimensions.
* Methods. Auxin Mediated Degradation. Was Auxin used in all the experiments? Or just ones that established the method?
* Supplemental tables - please ensure these are either in xlsx format or that there is a "wrap" specification. The content is missing at the end of each cell.

Curiosities
* Does this study give any insight into the fractional representation of erm-1a versus erm-1b?
* Could the speed of translational spots or their behaviors be useful in hypothesizing the mode of trafficking? For example, in Figure 4, would it be informative to plot the motion of the two differing translation spots on a kymograph? It may give some indication of how the speed of the translation spot compares to other trafficking processes such as microtubule-dependent motors. Alternatively, is there some other way to report or quantify the range of speeds?
* Figure 1G: 72 PP7 stem loops? That is a lot!

<u>Reviewer 3</u>

The current paper describes a substantial advance of the existing SunTag technique to image translation live. I am confident that it will have a positive impact on developmental biology. However, the data presented does not convince me that localized translation of erm-1 is important for EMR-1 function in the worm. This issue shouldn't necessarily preclude publication of the study as a techniques article. It has the potential to be a nice methods paper if the authors revise it in one of two ways. They either need to heavily qualify their conclusions regarding the biological significance of localized translation of erm-1 (in the title, abstract and main text). Or they need to provide new data to support their conclusions.

The article does a really nice job of going through how to get the SunTag system that was developed in cultured mammalian cells to work effectively in living worms - a powerful system for answering various developmental questions. Figuring out how to get an in vitro system to work in vivo was non-trivial (as is often the case), and the authors clearly and frankly discuss the challenges they faced and how they overcome these. Anyone with an interest in visualizing translation dynamics in worms can now build on this paper to answer their specific question.

Major Point

1) My major point relates to the conclusion that localized translation of erm-1 is important for EMR-1 function in the worm. This conclusion is based on the experiment where the authors ectopically localized erm-1 mRNA to the nuclear pore (through the combination of PP7 mRNA hairpins and a PCP binder fused to NPP-9) and then observed a partially penetrant defect in intestine morphology. However, a significant phenotype is already observed when only the PP7 loops are added to erm-1, even without changing its localization. Intestinal defects show up in about half the embryos (Fig 5E), the larva are much shorter (Fig S3E) and the worms are less fertile (FigS3D). Hence the introduction of the PP7 loops alone sensitizes the system significantly. Once the PP7 tagged mRNA is localized to the NPC the intestine phenotype does get worse on average, but the effect is quite modest in most individuals.

The addition of the PP7 loops to erm-1 clearly creates a hypomorph. In this background targeting the mRNA to NPC can exacerbate the hypomorph phenotype for reasons that don't inform on the significance of localized translation in wild type worms. For example, one possibility is that

© 2025. Published by The Company of Biologists under the terms of the Creative Commons Attribution License (https://creativecommons.org/licenses/by/4.0/).

ectopically tethering the erm-1 mRNA to the NPC could slightly decrease the levels of gene expression (translation doesn't normally happen here), which would then have comparatively large phenotypic effect in the context of a hypomorph, even though the same slight decrease in levels would be of no consequence in the wild type context.

To convincingly show that localized translation of erm-1 is important for EMR-1 function in the worm, it needs to be demonstrated in a context that isn't a hypomorph. Or at a bare minimum the authors should rule out the possibility that targeting mRNA to the NPC reduces levels of the protein it encodes. This can be tested with worms already created in this paper. Specifically by comparing the intensity of BFP from the eft-3p::24xGCN4::AID::T2A::BFP::H2B:20xPP7:tbb-2 3'UTR line when the mRNA is tethered to the NPC or not.

Minor Points

1) There appear to be several typos in the legend of figure 2. On line 686 "subjective" should be "subjected to" or some equivalent. A word seems to be missing on line 689, "Dunn's … test was used to assess statistical against", which doesn't make sense. Line 699 reads "… mRNAs may be underestimated due low labelling efficiency ...", this should presumably be "… due [to] low labelling efficiency …".

2) I think changing some of the color choices could make the figures easier to understand quickly. For example, in figure 1 G&H the schematics shows the scFv in green with the reporter gene encoding TagBFP in blue. However, in the next set of panels (1I-K) that show translation data it's shown in light blue which makes it unclear when you look quickly if it's mature proteins, which had been shown before in blue, or the scFv which had been shown in green. If the translation data is shown in the same color as it is shown in the schematics it would just make everything much clearer.

First revision

Author response to reviewers' comments

Comments from the Reviewers:

Reviewer 1: SUMMARY OF THE ADVANCE MADE IN THIS PAPER AND ITS POTENTIAL SIGNIFICANCE TO THE FIELD

The manuscript by van der Salm et al reports on the adaptation of the SunTag system for tracking co- translational targeting in C. elegans and apply this technique to understand the mechanisms and role of co-translational targeting and localized translation of the protein ERM-1.
As a general resource, it should be noted that the general principles of the system, including AID degradation have been developed in other systems in prior work. That said, the adaptation and optimisation of the system and demonstration of proof of principle in worms make it a potentially valuable resource for the community. I have a few minor comments in this regard and in general find the validation of the tool well supported.

Regarding ERM-1, the report provides a nice example of how the use of live imaging approaches enabled by the SunTag provide a much richer and dynamic picture of erm-1 translation. erm-1 mRNA has previously been reported to colocalize with ERM-1 protein in C. elegans in a translation-dependent manner, suggesting a model in which the emergence of the N-terminal membrane binding FERM domain targets mRNA to the membrane, enabling localised translation. Using the SunTag system, the authors can demonstrate local translation and additionally report on heterogeneity and dynamics of localisation/translation. For example, their data suggest that translation is enriched at membranes, but is not apically enriched,

© 2025. Published by The Company of Biologists under the terms of the Creative Commons Attribution License (https://creativecommons.org/licenses/by/4.0/).

suggesting that apical enrichment occurs post-translationally. The heterogeneity such as switching between translated and non-translated states as well as between membrane and cytoplasmic states also raises interesting questions. Finally, the authors report defects in ERM-1 function (though not localisation) when they mislocalize mRNA (and thus translation) to the nuclear envelope, suggesting a link between translation localisation and function. However, as I note below I am less convinced of this final point.

Finally, I appreciate the extensive discussion on potential pitfalls and caveats in applying this approach so that readers have a good grasp of the pros/cons/limits of the system. This is particularly useful for a resource article.

SUGGESTIONS TO AUTHORS
Major comments

My primary concern relates to claims that localised translation of erm-1 is important for function. This conclusion relies on the use of an mRNA tethering strategy based on the hairpin / hairpin binding protein PP7/PCP which is used to mislocalize erm-1 mRNA to the nuclear envelope. Complicating the interpretation of these experiments is that the SunTag and PP7 tagging already induce some defects on their own independent of targeting. The authors acknowledge this issue and show some specific ERM-1 associated defects, but interpretations are nonetheless complicated. Some particular issues:

First, PP7/PCP (mistargeted) and PP7(tag controls) both show similar phenotypes, though the severity appears somewhat worse for PP7/PCP vs PP7 controls. In the case of Figure 5E, the frequency of defects is similar, though the degree of severity is significantly different. In the case of Figure 5G, it is unclear if the observed differences are significant (see comment below on numerical data) and even so are relatively minor.
As noted, inserting PP7 sites into the *erm-1* 3' UTR affects ERM-1 expression independently of re-localization, complicating the analysis. Unfortunately, existing systems for re-localizing mRNAs—and thus studying the role of localized translation—are limited and inherently require genetic modification of the mRNA of interest, limiting our possibilities on how to study the importance of localized translation.

Nevertheless, we aimed to assess the importance of localized translation in our system and we agree with the reviewer that this underscores the importance of thoroughly characterizing the phenotypes and appreciate the suggestions for this.

To better characterize consequences of re-localizing *erm-1* to the nuclear pore at both the organismal and molecular levels, and to further support our conclusion that translation at the membrane is important for ERM's function, we implemented the following approaches based on reviewer suggestions:

1. Reducing the number of PP7 sites – We generated strains with fewer PP7 sites (20x, 8x and 2xPP7) with the intention to minimize negative effects on ERM-1 expression and create healthier starting strains. While the 2xPP7 failed to re-localize, and was thus excluded from analysis, in animals in which we inserted 8 instead of 20 PP7 sites ERM-1 levels where less affected and animals where healthier (see below).
2. Assessing translation efficiency at the nuclear pore – We carefully measured translation kinetics at the nuclear pore to rule out the possibility that the nuclear environment itself is inherently unfavorable for translation.
3. Examining organismal-scale effects – We conducted a more quantitative analysis of the developmental defects in *erm-1* re-localization strains.
4. Investigating molecular-scale effects – We expanded our analysis of the molecular consequences for ERM-1, measuring its expression (new in revision), localization (new in revision), membrane dynamics (new in revision), and its ability to link the actin cytoskeleton to the membrane.

Specifically, to measure developmental defects more quantitatively, we shifted from categorical phenotyping to counting the number of abnormalities per embryo in both control and re-localized strains. This approach revealed a significant increase in intestinal abnormalities when *erm-1* was

© 2025. Published by The Company of Biologists under the terms of the Creative Commons Attribution License (https://creativecommons.org/licenses/by/4.0/).

translated at the nuclear pore rather than the membrane in 8xPP7 strains. While 20xPP7 strains showed a similar trend, it did not reach statistical significance. We believe this method provides a more accurate measure of developmental defects and effectively highlights significant differences between control and re-localized strains (8xPP7).

Second, both PP7 and PP7/PCP appear to show reductions in ERM expression suggesting the PP7 hairpins and potentially PCP binding to hairpins influence expression. Indeed, it appears that levels are lower in the PP7/PCP compared to PP7 alone, though quantification is noticeably lacking here.

The immunofluorescent stainings (IF) in our initial manuscript were highly variable, a well-known issue in *C. elegans* due to challenges in eggshell permeabilization. We felt that quantification would primarily reflect staining efficiency rather than expression levels. To address this, we re-created the PP7 strains in *ERM- 1::GFP* strains and quantified GFP expression in live animals in control and re-localized strains (see below).

Given these points, it is difficult to interpret the impact of targeting on phenotypes. Specifically:
- Are phenotypes simply due to expression differences?
To assess expression levels of ERM-1 upon inserting PP7 sites, we introduced PP7 hairpins into an *erm- 1::GFP* strain. Both 20x and 8xPP7 strains showed reduced ERM-1::GFP expression, consistent with the idea that inserting PP7 hairpins triggers mRNA decay (Fig. S3I,J). Notably, ERM-1 levels were more affected in the 20xPP7 strain than in the 8xPP7, likely due to the greater number of repeats and the resulting longer 3'UTR. In 8xPP7 strains expression levels remained comparable to those in heterozygous *erm-1::GFP* animals, which develop normally, suggesting that the observed lower expression levels are not the cause for the observed developmental defects.

Interestingly, in line with increased translation efficiency at the nuclear pore (Fig S3) and reporter observations, re-localization restored ERM-1::GFP levels closer to WT in 8x and 20xPP7 larvae and 20xPP7 embryos. However, this effect was absent in 8xPP7 embryos (Fig. S3I). The underlying cause remains

unclear but may be related to increased ERM-1 dynamics when synthesized at the nuclear pore (see Fig. 5G,H). Overall, while ERM-1 expression is affected by inserting PP7 levels, its levels appear sufficient for normal development (especially in the 8xPP7 strain), and *erm-1* translation efficiency is not lower at the nuclear pore, making these strains well suited for studying the functional consequences of disturbed localized translation.

- Is the phenotype due to mistargeting or simply to the PP7/PCP tethering per se?
- if reduced expression is specific to nuclear mistargeting, is this due to the lack of membrane targeting or more to the nature of the mistargeting, for example if the nuclear pore environment is problematic? The latter would be less interesting as it is unlikely to end up there?

To rule out the possibility that the nuclear environment is inherently unfavorable for translation, we first tethered our reporter mRNA to both the membrane and nuclear pore. Translation was efficient in both locations, with even higher efficiency at the nuclear pore compared to the membrane (Fig. S3D). Consistently, BFP expression was elevated when mRNA was tethered to the nuclear pore, suggesting the nuclear environment is an active site for translation (Fig. S3E,F).

Next, we measured *erm-1* translation efficiency at the nuclear pore. Nuclear pore-localized *erm-1* mRNAs were efficiently translated, with similar translation spot number and slightly increased intensity in re- localized *erm-1* strains compared to non-localized *erm-1 PP7* controls. These results indicate that translation at the nuclear pore is not reduced and does not underlie the observed developmental defects. Taken together with the more normal ERM-1 levels in 8xPP7 strain, this makes it highly unlikely that defects are related to reduced ERM-1 levels.

For the first point, more accurate quantification of expression differences, partial RNAi / heterozygotes, or overexpression would be informative here.

© 2025. Published by The Company of Biologists under the terms of the Creative Commons Attribution License (https://creativecommons.org/licenses/by/4.0/).

See also above. We compared ERM-1::GFP expression levels in strains in which we inserted PP7 sites in the 3' UTR of erm-1 to those in homozygous and heterozygous erm-1::GFP animals. Our analysis showed that ERM-1::GFP expression in the 8xPP7 strains is comparable to that in heterozygous erm-1::GFP animals (Fig S3I,J).

For the latter two points, it would be informative to compare the effects of distinct PCP-tethers, e.g. free PCP, plasma membrane, mitochondrial, nuclear. If plasma membrane targeting were critical for function, I would expect that free PCP and plasma membrane binding PCP should not enhance the phenotype of PP7 alone. In an ideal world, one would be able to disrupt the endogenous targeting signal (the FERM domain) and rescue via membrane PCP tethering, but as the FERM domain is critical for function, it is unclear how to approach this problem. Perhaps adding an mis-targeting domain to the SunTag sequence before the T2A to compete with the FERM domain. That said, the use if different PCP-tethers may be sufficient.

The idea of altering *erm-1*'s translation site without disrupting its function is excellent. However, as the reviewer noted, mutations that shift translation also affect *erm-1* function, making it nearly impossible to separate the effects of translation site disruption from those on protein activity.

To evaluate the impact of tethering mRNAs to different cellular locations, we first tethered our reporter to both the membrane and the nuclear pore. In both cases, tethering was efficient, with translation appearing more robust at the nuclear pore than at the membrane (Fig. S3A-F). Over the past months, we attempted a similar approach for *erm-1*, aiming to tether it to the membrane by expressing a PCP::mCherry::PH construct. However, technical challenges with genetic crosses and developmental issues caused by extra- chromosomal *PCP::mCherry::PH* overexpression prevented us from successfully generating these lines.

As a result, we cannot directly compare the effects of tethering alone to those of tethering *erm-1* to the nuclear pore. Nonetheless, our findings show that nuclear pore tethering does not reduce *erm-1* translation—and may even enhance it—suggesting that the observed phenotypes arise from the requirement for *erm-1* translation at the membrane rather than from PCP binding.

Additional minor comments
I am not an expert in this area, but the SunTag is built around the GCN4 leucine zipper. Does it show intrinsic di-/oligomerisation and would this impact interpretation?

The SunTag system relies on GCN4 peptides to recruit antibodies. This translation-imaging method has been widely used across different systems without reported issues of oligomerization. We have no evidence suggesting it occurs in our system either.

I may be misunderstanding something, but in Figure 2 and associated text, it could be clearer why the membrane targeting of mRNA via the PP7/PCP system was important for demonstrating coincidence of mRNA and the SunTag reporter. Presumably one could look at colocalization of mRNA and the SunTag without this. I presume it aided tracking, but was unclear.

Thank you for the suggestion. Tethering mRNAs indeed facilitates long-term visualization of single molecules, which would otherwise diffuse rapidly and move out of focus. We have added a sentence to clarify this in the manuscript (line 165).

Line 188: "We observed a slightly slower growth rate..." Does this refer to Figure S3E?

We have added the correct reference to the figure

Figure S2B, Line 197: Cites translation being enriched in intestinal precursors. This is not critical for the paper, but given the fact that only one embryo is shown, there are relatively few spots, and no quantification, I don't think the authors can draw a conclusion here.

We have updated the text (line 195).

© 2025. Published by The Company of Biologists under the terms of the Creative Commons Attribution License (https://creativecommons.org/licenses/by/4.0/).

Figure 5E, 5G: It would be helpful for readers to have access to the underlying numerical data for these frequency plots to judge the significance of the relevant differences. Particularly in the case of Figure 5G, could the differences be expected based on chance alone? How robust are the frequencies across replicates? Note for completeness, Figure S3B also lacks underlying numerical data, but the differences here are so large I am less worried.

Thank you for your suggestion to again look at how to quantify the developmental defects (originally Fig. 5E, now Fig. 5F). In the plots shown in the initially submitted manuscript we categorized all imaged animals as severe, medium, mild or WT. We here pooled all the data from different experiments, so there were no replicates. To measure developmental defects more quantitatively, we shifted from categorical phenotyping to counting the number of abnormalities per embryo in both control and re-localized strains. This approach revealed a significant increase in intestinal abnormalities when *erm-1* was translated at the nuclear pore rather than the membrane in 8xPP7 strains. While 20xPP7 strains showed a similar trend, it did not reach statistical significance. We believe this method provides a more accurate measure of developmental defects and effectively highlights significant differences between control and re-localized strains (8xPP7).

Figure S3C (Differences in expression): Quantification missing.

We replaced this figure with one measuring GFP levels in live animals, instead of immunohistochemical stainings that are highly variable. The latter is a well-known issue in *C. elegans* research, caused by challenges in eggshell permeabilization. Therefore, we felt that quantification of immunostainings would primarily reflect staining efficiency rather than expression levels. To address this, we re-created the PP7 integrations in *ERM-1::GFP* strains and quantified GFP expression in live animals (Fig. S3I, J).

Reviewer 2: This manuscript develops a novel nascent peptide imaging system that can be used to assess translation dynamics in real time and at the single-molecule level in C. elegans. The authors apply their technology to the study of ERM-1, a conserved membrane-linker protein crucial for cell function and development. Previous work had shown the erm-1 mRNA and protein both co-localize at the plasma membrane in a translation-dependent manner. However, the dynamics of the process and their functional relevance could not be assessed using previous methods.

Here, the authors leverage innovative live imaging approaches to address both questions. Using an adapted SunTag system, they discover ERM-1 translation does indeed occur at the membrane and that its behavior is strikingly dynamic and heterogenous. The group next generated a method to artificially tether the erm-1 mRNA to an ectopic locale in the cell. This resulted in developmental defects that were specifically associated with the ectopic tethering. Therefore, they verified that the site where ERM-1 is translated in the cell impacts its function.

This paper makes several significant and novel contributions to our understanding. #1) It develops translational imaging technology in C. elegans, a breakthrough feat. #2) It introduces an ectopic mRNA tethering approach in C. elegans that has the potential to be adapted for other transcripts. #3) It illustrates that ERM-1 is locally translated as hypothesized with some surprising behaviors. #4) It illustrates that ERM-1's local translation is required for its function.

The value of this last contribution cannot be understated. Multiple mRNAs in C. elegans have been shown to have translation-dependent mRNA localization. This is interesting but the functional relevance was heretofore unestablished. Frustratingly, protein motifs required for localization are typically also crucial for function, preventing of ablation of one without the other. The PP7 approach adapted here circumvents this technical limitation and can be used in the study of other transcripts.

Other highlights of the paper:
* **The authors find that erm-1 mRNA may release from the membrane after completion of one**

© 2025. Published by The Company of Biologists under the terms of the Creative Commons Attribution License (https://creativecommons.org/licenses/by/4.0/).

round of translation and then re-associate at the next round. This is an unexpecting finding that underscores the value of a live imaging approach.
* **The paper quantifies how PP7 stem loops can lead to disrupted mRNA behavior**
* **There are several elegant controls utilized**
* **The shortcomings and limitations are clearly described**

Overall, I have only one Major Concern, a few Mid-Level Concerns, and a list of completely optional Minor Suggestions and General Curiosities. All are listed below.
In sum, I recommend this paper for publication in Development with minor revisions.

Major Concern
* **My major concern may be a limitation of the submission process. The paper needs movies to illustrate the dynamical movements of ERM-1. Please include a selection of movies for the readers to evaluate.**

Thank you for the suggestion. We have included a movie as supplemental file Mov S1
Mid-level concerns
* **What is the logic that fully translated proteins are dimmer than the actively translated protein? Is the idea that they are undergoing auxin-mediated decay? If so, do they disappear? Or do they remain dim?** Because multiple ribosomes simultaneously translate a single mRNA and each ribosome produces a nascent peptide chain that can be bound by multiple antibodies—up to 24 per nascent peptide chain— this results in the recruitment of numerous antibodies per translating mRNA, which appears as a bright fluorescent spot under the microscope. Although mature proteins are also labeled by the SunTag system, their fluorescence is much dimmer, as each protein can bind at most 24 antibodies. We have also clarified this now in the text, line 103. Moreover, when combined with the AID system the mature proteins indeed disappear after translation, while the nascent peptide chains bound by ribosomes remain unaffected during translation.

* **Line151. Consider rewording to avoid the word "termination" here as it implies translational termination. What about "translational repression", "stress", or "heat stress" - terms that more accurately reflects the process.**
Thank you for the suggestion, we changed termination to inhibition (now line 146)

* **Figure 2B. Why was this particular statistical test chosen? Maybe include a statement like, "...to account for differing numbers of replicates" to explain the departure from the Welch's t-test that was used in the other parts of the paper.**
We had chosen the Welch’s t-test for figures 1F and 2D as we compared two populations of normally distributed data. We had chosen the Kruskal-Wallis test with Dunn’s multiple comparison post-hoc analysis for figure 2B as 1) the data did not pass our D’Agostino-Pearson test for normality and 2) more than two populations were compared to each other. These characteristics do in our opinion not fit a Welch’s t-test. We chose for the Kruskal-Wallis test as we aimed to perform all statistical analysis in a similar pre- determined manner, as described in the methods section under *Statistical analysis*.
* **Figure 2D. Can you clarify the Y-axis either in the figure or legend or both? The way it is written as "overlap", it is unclear whether this is the percent of translation spots that contain mRNA or the percentage of mRNA spots that contain translation. Something like "Overlap spots as a fraction of the total translation spots".**
Thank you, we have now changed the labeling of the axis to ‘Translation spots overlapping with mRNA spots (%).
* **Can you clarify the idea that there is germline silencing happening in early embryos? Or, is the eft-3 promoter not transcribed until the 16-cell stage? If the reporter is silenced in early embryos, how are the 2-cell and 4-cell images in Fig 1C - F achieved?**
This silencing of the reporter is likely caused by the repetitive nature of the 24xSunTag peptide array, as the SunTag antibody (also driven by an *eft-3* promotor) is expressed in the early embryos (Fig. 1C). Endogenous proteins tagged with only 1 or 2 SunTag peptides (Fig. 1D-F) are expressed normally in the early embryo.

* **Page 5. Line 158 - 164. The authors state that translation spots that do not have a**

© 2025. Published by The Company of Biologists under the terms of the Creative Commons Attribution License (https://creativecommons.org/licenses/by/4.0/).

corresponding mRNA probe spot must indicate that the smiFISH technology has low labeling efficiency. Low labeling efficiency may play a role, but how does one distinguish low labeling efficiency from a fully translated peptide, one that has dissociated from the mRNA but has not yet degraded?
Yes, this is indeed also possible, although individual mature proteins should be less bright. To clarify this, we added the following sentence to the document: This could have two possible explanations. First, some identified bright GFP spots may not represent active translation sites, but rather mature proteins or protein aggregates that met our analysis threshold. Second, this may reflect a low labeling efficiency of our mRNA probes (lines 154)
* **How large is the GCN4 protein/peptide? Is this a protein from yeast? Or is this a shortened peptide? Some indication of the length and sequence would be helpful to envision the size of the N-terminal tag.**
The GCN4 tag is 19 amino acids, plus a linker between the peptides. The 24xGCN4 array is 1,8kb. We have added this to the text both in the section where we explain the SunTag system and in the discussion (lines 115, 122 and 407)

* **On that note, wild-type ERM-1 requires a functional N-terminal FERM domain for localization. Because the GCN4 tags are placed N-terminal to the FERM tag, it is possible that any time lapse or pauses in translation between the GCN4 translation and FERM domain may lead to a delay in localization to the membrane. This may account for some fraction of the cytoplasmic translating mRNAs. Specifically, mention this caveat on Pg. 7 Lines 231 - 232.**
You are correct that it will take some time for the first ribosome to reach the PIP2 binding domain. However, since 5 minutes should be sufficient for ribosomes to reach the PIP2 binding domain in the *erm-124xSunTag* strain, we would expect that if every translating ribosome causes *erm-1* mRNA to localize to the membrane, we should see all mRNAs localizing to the membrane within the 5 minutes time frame of our movie. We have added a sentence explaining that 'this suggests that not every nascent peptide will interact with the membrane, preventing to mRNA localization to the membrane' (line 231), and further discussed this in the discussion (line 400).

* **How does the addition of GCN4 and/or PP7 sequences impact mRNA abundance?**
We have not performed smFISH with and without PP7 sites. However, one likely reason for the lower ERM- 1 expression upon inserting PP7 sites is PP7-triggered mRNA decay and we expect to see fewer mRNAs in strains in which PP7 was inserted into *erm-1*. This is also in line with the observation that inserting PP7 sites reduces the number of translation spots (Fig. S3C). Regarding the insertion of GCN4 repeats, we observed that ERM-1 expression is silenced in the early embryo, resulting in lower mRNA levels. Later, no differences were observed in mRNA levels between *erm-1::GFP* and 24*GCN4::ERM-1* (Fig S2).

Minor Points
* **Fig 1D,E are referenced in opposite order in the figure and in the text.**
Thank you for noticing, we changed it.
* **Figure 1 Legend. 1K. The Figure legend for 1K references two figures, but only one is shown.**
Thank you for noticing, we changed this.

* **Figure 2D figure legend. Line 702. "where" should be "were"'.**
Thank you for noticing, we changed it.

* **Page 7. Line 237. Missing period after the word ERM-1.**
Thank you for noticing, we added the period.

* **Figure 4A. Could you label the dotted rectangles that indicate insets as either "B" or "C"? It will aid the reader in.**
We implemented your suggestion in the revised manuscript.

* **Page 8. Line 262. In this section, could you highlight that there are no other copies of erm-1 in the strain?**
As a result of the revised experiments, large parts of the text have been changed in this section. In the new text, we have highlighted this in line 271. As depicted in Fig. 3A, there are two copies of *erm-1* (*erm- 1a* and *erm-1b*) and we have only integrated the 24xGCN4 repeats in *erm-1a*. However, both *erm-1a* and *erm-1b* have the same 3'UTR and both contain PP7 integrations for re-

© 2025. Published by The Company of Biologists under the terms of the Creative Commons Attribution License (https://creativecommons.org/licenses/by/4.0/).

localization to the nuclear pores

* **Page 12. Line 406 - Again, how big is each GCN4 unit? Also, this explanation doesn't account for any pauses in translation that may occur.**
We added the information about the size and further clarified this point in line 407.

* **Figure 3C. What is randomized here? The GCN sequence? Or was it a random, computational selection of X, Y, Z coordinates?**
The randomized control was generated by flipping only the scFv::GFP signal with respect to the Z-axis to create a random distribution of translation spots while maintaining most translation spots within the embryo marked by the mCherry::PH (see method section: quantification of localized translation).

* **Figure 5C. can you clarify the Z-stacking here? Is it single slice, a few slices, or max project?**
It is a maximum projection of 19 Z-stacks.

* **Methods: Progeny size. Should this be "Brood Size"? Progeny size implies a measurement of worm dimensions.**
Thank you, we implemented this suggestion.
* **Methods. Auxin Mediated Degradation. Was Auxin used in all the experiments? Or just ones that established the method?**
Auxin was used for strains expressing the AID system. We have added information to the methods section to clarify the use of auxin.

* **Supplemental tables - please ensure these are either in xlsx format or that there is a “wrap” specification. The content is missing at the end of each cell.**
Yes, we have added the correct tables in the revised manuscript.

Curiosities
* **Does this study give any insight into the fractional representation of erm-1a versus erm-1b?**
That's an interesting point, which isn't specifically addressed in this work. However, from ongoing experiments in the lab, outside the scope of this project, we know that *erm-1a* is the main isoform expressed in the germline and early embryo. This is relevant because our focus is primarily on the early embryo, and we have tagged only *erm-1a* with the SunTag peptides. Since the PP7 sites are inserted in the 3' UTR, which is shared by both *erm-1a* and *erm-1b*, both isoforms are relocated to the nuclear pore, making no distinction between *erm-1a* and *erm-1b* in these experiments.
* **Could the speed of translational spots or their behaviors be useful in hypothesizing the mode of trafficking? For example, in Figure 4, would it be informative to plot the motion of the two differing translation spots on a kymograph? It may give some indication of how the speed of the translation spot compares to other trafficking processes such as microtubule-dependent motors. Alternatively, is there some other way to report or quantify the range of speeds?**
Tracking the speed and dynamics of individual translation sites is highly valuable. So far, we have only tracked a limited number of spots, which is not sufficient to distinguish different populations. In the future, a more extensive analysis, which may require the development of an automated pipeline, could provide valuable insights in the mean square displacement and diffusion constants, helping to characterize the mobility and potential interactions of translating mRNAs within the cell. We would love to explore this further.

* **Figure 1G: 72 PP7 stem loops? That is a lot!**
We agree. It is an optimized construct in which the linkers between the stem loops have been shortened, so that the total size length is limited as much as possible. In the revised manuscript we showed that 8xPP7 sites are sufficient to re-localized mRNAs. However, visualization of single mRNAs required more stem loops.

Reviewer 3: The current paper describes a substantial advance of the existing SunTag technique to image translation live. I am confident that it will have a positive impact on developmental biology. However, the data presented does not convince me that localized translation of erm-1 is important for EMR-1 function in the worm. This issue shouldn't

© 2025. Published by The Company of Biologists under the terms of the Creative Commons Attribution License (https://creativecommons.org/licenses/by/4.0/).

necessarily preclude publication of the study as a techniques article. It has the potential to be a nice methods paper if the authors revise it in one of two ways. They either need to heavily qualify their conclusions regarding the biological significance of localized translation of erm-1 (in the title, abstract and main text). Or they need to provide new data to support their conclusions.

The article does a really nice job of going through how to get the SunTag system that was developed in cultured mammalian cells to work effectively in living worms - a powerful system for answering various developmental questions. Figuring out how to get an in vitro system to work in vivo was non-trivial (as is often the case), and the authors clearly and frankly discuss the challenges they faced and how they overcome these. Anyone with an interest in visualizing translation dynamics in worms can now build on this paper to answer their specific question.

Major Point

1) My major point relates to the conclusion that localized translation of erm-1 is important for EMR-1 function in the worm. This conclusion is based on the experiment where the authors ectopically localized erm-1 mRNA to the nuclear pore (through the combination of PP7 mRNA hairpins and a PCP binder fused to NPP-9) and then observed a partially penetrant defect in intestine morphology. However, a significant phenotype is already observed when only the PP7 loops are added to erm-1, even without changing its localization. Intestinal defects show up in about half the embryos (Fig 5E), the larva are much shorter (Fig S3E) and the worms are less fertile (FigS3D). Hence the introduction of the PP7 loops alone sensitizes the system significantly. Once the PP7 tagged mRNA is localized to the NPC the intestine phenotype does get worse on average, but the effect is quite modest in most individuals.

The addition of the PP7 loops to erm-1 clearly creates a hypomorph. In this background targeting the mRNA to NPC can exacerbate the hypomorph phenotype for reasons that don't inform on the significance of localized translation in wild type worms. For example, one possibility is that ectopically tethering the erm-1 mRNA to the NPC could slightly decrease the levels of gene expression (translation doesn't normally happen here), which would then have comparatively large phenotypic effect in the context of a hypomorph, even though the same slight decrease in levels would be of no consequence in the wild type context.

To convincingly show that localized translation of erm-1 is important for EMR-1 function in the worm, it needs to be demonstrated in a context that isn't a hypomorph. Or at a bare minimum the authors should rule out the possibility that targeting mRNA to the NPC reduces levels of the protein it encodes. This can be tested with worms already created in this paper. Specifically by comparing the intensity of BFP from the eft-3p::24xGCN4::AID::T2A::BFP::H2B:20xPP7:tbb-2 3'UTR line when the mRNA is tethered to the NPC or not.

We understand the concern that the developmental defects in *erm-1* re-localization mutants may result from the control strain being hypomorphic rather than reflecting a genuine requirement for *erm-1* translation at the membrane. Indeed, inserting PP7 sites into the *erm-1* 3’ UTR affects ERM-1 expression independently of re-localization, complicating the analysis.

Unfortunately, existing methods for re-localizing mRNAs—and studying the role of localized translation— are inherently limited, as they require genetic modifications that can impact mRNA biology. While these technical constraints prevent us from generating a completely unaffected strain while simultaneously addressing localized translation, we implemented three strategies to mitigate these concerns

1. Reducing the number of PP7 sites, which resulted in healthier starting strains
2. Assessing translation efficiency at the nuclear pore
3. Expanding our analysis of the molecular consequences of *erm-1* re-localization

© 2025. Published by The Company of Biologists under the terms of the Creative Commons Attribution License (https://creativecommons.org/licenses/by/4.0/).

1. Reducing the number of PP7 sites

Since inserting multiple PP7 sites causes general defects, we generated *erm-1* strains with fewer insertions to create less affected control strains. In addition to our 20xPP7 strain, we created 8xPP7 and 2xPP7 strains. Both 20xPP7 and 8xPP7 *erm-1* mRNAs efficiently re-localized to the nuclear pore upon PCP::mCherry::NPP- 9 expression (71% and 74%, respectively), whereas 2xPP7 failed to re-localize (only 30% was re-localized) and was excluded from further analysis.

Quantification of ERM-1::GFP levels revealed that ERM-1::GFP was less affected in 8xPP7 animals than in the 20xPP7 strain, with expression levels comparable to heterozygous *erm-1::GFP* animals, which develop normally. Additionally, larval defects in 8xPP7 strains were milder than in 20xPP7 (Fig. S3L), indicating that (1) we successfully generated a healthier strain and (2) ERM-1::GFP levels in our re-localization strain are similar to those in normally developing heterozygous *erm-1* animals. Why the 8xPP7 strain still exhibits some developmental defects is unclear.

While an entirely unaffected starting strain would be ideal, the 8xPP7 strain provides a solid foundation for investigating the effects of translation re-localization. However, since 8xPP7 animals still exhibit some developmental defects, we cannot fully exclude the possibility that the sensitized background exacerbates the observed phenotypes. Nevertheless, even if this is true, the most likely explanation for our observed results is that the molecular consequences of *erm-1* re-localization—such as disrupted localization, increased dynamics, and impaired membrane-actin linkage—arise directly from translation site disruption.

2. *Assessing Translation at the Nuclear Pore*

To rule out the possibility that the nuclear environment is inherently unfavorable for translation, we first tethered our reporter mRNA to both the membrane and nuclear pore. Translation was efficient in both locations, with even higher efficiency (intensity of the translation spots) at the nuclear pore (Fig. S3D). Consistently, BFP expression was elevated when mRNA was tethered to the nuclear pore, suggesting the nuclear environment is an active site for translation (Fig. S3E,F).

Next, we measured *erm-1* translation efficiency at the nuclear pore. Nuclear pore-localized *erm-1* mRNAs were efficiently translated, with similar translation spot number and slightly increased intensity in re- localized *erm-1* strains compared to non-localized *erm-1 PP7* controls. These results indicate that translation at the nuclear pore is not reduced and does not underlie the observed developmental defects. Taken together with the more normal level in 8xPP7 strain, this makes it highly unlikely that defects are related to ERM-1 levels.

3. *Expanding Molecular Analyses*

To further investigate the molecular consequences of *erm-1* re-localization, we expanded our analysis beyond developmental defects. We examined *erm-1* expression levels (Fig. S3I,J), localization (Fig. 5H,J), ERM-1 dynamics at the membrane (Fig. 5G,I), and its role in linking the actin cytoskeleton to the membrane. While PP7 site insertion reduces overall ERM-1 levels and causes some general defects (Fig. S3), re- localization specifically disrupts membrane targeting, increases ERM-1 turnover, and impairs actin recruitment to apical sites. These findings support a model in which the site of translation directly impacts ERM-1 behavior, with functional consequences for ERM-1 activity and lumen formation.

Minor Points

1) There appear to be several typos in the legend of figure 2. On line 686 "subjective" should be "subjected to" or some equivalent. A word seems to be missing on line 689, "Dunn's … test was used to assess statistical against", which doesn't make sense. Line 699 reads "… mRNAs may be underestimated due low labelling efficiency ...", this should presumably be "… due [to] low labelling efficiency …".

Thank you for noticing, we have updated the text to include your suggestions.

2) I think changing some of the color choices could make the figures easier to understand quickly. For example, in figure 1 G&H the schematics shows the scFv in green with the reporter gene encoding TagBFP in blue. However, in the next set of panels (1I-K) that show translation data it's shown in light blue which makes it unclear when you look quickly if it's mature proteins, which had been shown before in blue, or the scFv which had been shown in green. If the translation data is shown in the same color as it is shown in the schematics it would just

© 2025. Published by The Company of Biologists under the terms of the Creative Commons Attribution License (https://creativecommons.org/licenses/by/4.0/).

make everything much clearer.

Thank you for your suggestions. We have changed the schematics to match the color of the translation spots depicted in the figures.

Second decision letter

MS ID#: dev.204435R1

MS TITLE: Measuring and manipulating localized translation of erm-1 in the C. elegans embryo

AUTHORS: Elise van der Salm; Esther Koelewijn; Mette Schroeder; Erica van der Maas; Olga Jarosińska; Max Eeken; Suzan Ruijtenberg

Dear Dr Ruijtenberg,

I have now received all the referees reports on the above manuscript, and have reached a decision. The referees' comments are appended below, or you can access them online: please go to .

The overall evaluation is positive and we would like to publish a revised manuscript in Development. However, the reviewers provide additional comments to further improve the clarity of the paper. Please attend to all of the reviewers' comments in your revised manuscript and detail them in your point-by-point response. If you do not agree with any of their criticisms or suggestions explain clearly why this is so.

Reviewer 1

The revised version by van der Salm et al has largely addressed my concerns. In particular:

- The quantification of defects between PP7 and PP7+PCP are more rigorously assessed. Quantification is improved throughout.
- They have shown that reduced expression is unlikely to be the root cause of defects. This includes quantitative analysis of expression using GFP::ERM-1 variants as well as showing that nuclear envelope is permissive for expression of mistargeted mRNA.
- They have also attempted to minimise the effect of PP7 insertions by creating variants with fewer sites, which reduces the background phenotypes and allows them to better distinguish the effects of mistargeting.

I still have some reservations about the conclusion that targeted translation is important for ERM function. It remains possible that it is the tethering per se, rather than location of tethering that underlies the phenotypes as they were unable to generate the lines required to tether ERM-1 to the plasma membrane. The effects are also relatively modest (and not seen in 20xPP7+PCP presumably because of the high baseline of defects of 20xPP7). That said, it is important to note that they do see significant effects, at least in the embryo, and I think the authors have made reasonable efforts to solidify their findings.

Given that the adaptation and benchmarking of the method should be a valuable resource for the community and in light of the additional data in support of their observations, combined with a balanced discussion and revised title, I support publication.

I have a few additional comments:

Line 290: Strikingly, animals in which erm-1 was translated at the nuclear pore showed several
291 developmental defects, resembling erm-1 mutants, including cysts and constrictions in the intestinal lumen in
292 both embryos (Fig. 5F) and larvae (Fig. S3K, L).

© 2025. Published by The Company of Biologists under the terms of the Creative Commons Attribution License (https://creativecommons.org/licenses/by/4.0/).

Unless I am misreading things, there appears to be no difference between PP7 and PP7+PCP for these experiments, hence most of the effect on larva appears to be accounted for by PP7 alone. Hence, I would omit reference to larval phenotypes here.

Line 305: This aligns with findings from phosphorylation mutants, where reduced apical enrichment correlates with increased ERM-1 turnover at the membrane (Ramalho et al., 2020).

Worth nothing that the effects here in FRAP are very small compared with those reported in Ramalho. Also, the text cites changes in recovery and immobile fractions from these experiments, but I could not find any quantification of this or how the data was fit. Based on the recovery timescale and the near linear nature of the curves, I would expect the fits to have quite a bit of uncertainty when fit to a simple FRAP model. Finally, it was unclear to me what values were being compared in the statistics (Anova).

Reviewer 3

While I'm still not convinced that the localized translation of erm-1 is important in wild type worms, the revisions have significantly improved the paper, and it will be a useful resource for the worm community.

Second revision

Author response to reviewers' comments

Point-by-point response

Reviewer 1:

Line 290: Strikingly, animals in which erm-1 was translated at the nuclear pore showed several developmental defects, resembling erm-1 mutants, including cysts and constrictions in the intestinal lumen in both embryos (Fig. 5F) and larvae (Fig. S3K, L). Unless I am misreading things, there appears to be no difference between PP7 and PP7+PCP for these experiments, hence most of the effect on larva appears to be accounted for by PP7 alone. Hence, I would omit reference to larval phenotypes here.

- *We have removed the reference to the larval phenotypes from this line and moved it to the first sentence of the paragraph (line 289).*

Line 305: This aligns with findings from phosphorylation mutants, where reduced apical enrichment correlates with increased ERM-1 turnover at the membrane (Ramalho et al., 2020). Worth noting that the effects here in FRAP are very small compared with those reported in Ramalho. Also, the text cites changes in recovery and immobile fractions from these experiments, but I could not find any quantification of this or how the data was fit. Based on the recovery timescale and the near linear nature of the curves, I would expect the fits to have quite a bit of uncertainty when fit to a simple FRAP model. Finally, it was unclear to me what values were being compared in the statistics (Anova).

- *We have indicated that the measured differences in FRAP recovery in the re-localization strains are smaller than the previously observed effects with the phosphomutants (line 304)*
- *We removed 'and a higher mobile fraction' from (line 303).*
- *We have clarified how we performed the statistical analysis on the FRAP data in the 'statistical analysis' section (line 655)*
- *Finally, we have added 'MetaMorph Microscopy Automation & Image Analysis Software' to*

© 2025. Published by The Company of Biologists under the terms of the Creative Commons Attribution License (https://creativecommons.org/licenses/by/4.0/).

the list of software programs used for imaging and image analysis to the section ' Microscopy and Image analysis' (line 515)

Third decision letter

MS ID#: dev.204435R2

MS TITLE: Measuring and manipulating localized translation of erm-1 in the C. elegans embryo

AUTHORS: Elise van der Salm; Esther Koelewijn; Mette Schroeder; Erica van der Maas; Olga Jarosińska; Max Eeken; Suzan Ruijtenberg
ARTICLE TYPE: Techniques and Resources Article

I am happy to tell you that your manuscript has been accepted for publication in Development, pending our standard publication integrity checks.

© 2025. Published by The Company of Biologists under the terms of the Creative Commons Attribution License (https://creativecommons.org/licenses/by/4.0/).
